# Dirhamnolipid ester – formation of reverse wormlike micelles in a binary (primerless) system

**DOI:** 10.3762/bjoc.16.232

**Published:** 2020-11-19

**Authors:** David Liese, Hans Henning Wenk, Xin Lu, Jochen Kleinen, Gebhard Haberhauer

**Affiliations:** 1 Institut für Organische Chemie, Universität Duisburg-Essen, Universitätsstraße 7, D-45117 Essen, Germany; 2 Evonik Operations GmbH, Evonik Industries AG, Goldschmidtstraße 100, D-45139 Essen, Germany

**Keywords:** dirhamnolipid ester, gemini surfactant, rheology, reverse wormlike micelle (RWLM)

## Abstract

We report new dirhamnolipid ester forming reverse wormlike micelles in nonpolar solvents without the addition of any primer. Therefore, these compounds represent a rare case of a binary system showing this gel-like behavior. In this study, the influence of the concentration of the rhamnolipid ester and the ester alkyl chain length on the rheological properties of the reverse wormlike micelles in toluene was investigated in detail. Highly viscoelastic solutions were obtained even at a relatively low concentration of less than 1 wt %. The phase transition temperatures indicate that the formation of reverse wormlike micelles is favored for dirhamnolipid esters with shorter alkyl chain lengths. Oscillatory shear measurements for the viscoelastic samples reveal that the storage modulus (*G'*) and the loss modulus (*G''*) cross each other and fit the Maxwell model very well in the low-ω region. As is typical for wormlike micelle systems, the normalized Cole–Cole plot of *G''*/*G''*_max_ against *G'*/*G''*_max_ was obtained as a semicircle centered at *G'*/*G''*_max_ = 1. The formation of network structures was also verified by polarized light microscopy. The sample was birefringent at ambient temperature and anisotropic at an elevated temperature. Differential scanning calorimetry analysis yielded a transition enthalpy of about Δ*H*_SG/GS_ = ±7.2 kJ/mol. This value corresponds to a strong dispersion energy and explains the formation of the highly viscous gels by the entanglement of wormlike micelles through the interaction of the alkyl chains.

## Introduction

**Figure 1:**
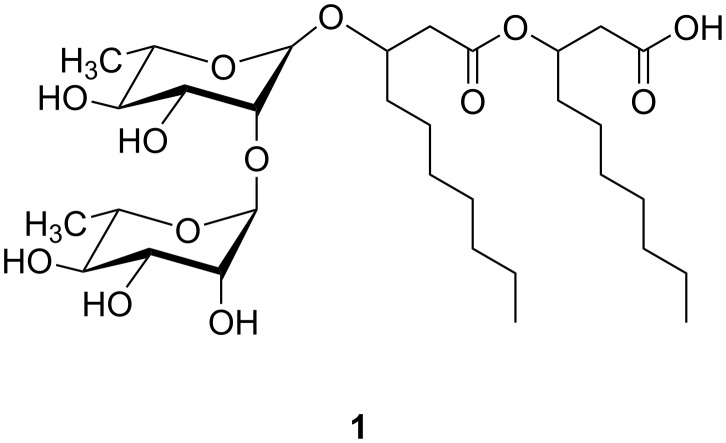
Chemical structure of dirhamnolipid **1**.

Surfactants have both hydrophilic and hydrophobic groups and are therefore amphiphilic molecules. Due to their unique molecular structure, surfactants are essential ingredients in a lot of technical applications. They can act as a flotation agent in the enrichment of ores [1], emulsifier and stabilizer for emulsions [2], or as additives for self-cleaning surfaces (artificial lotus effect) [3–4]. Rhamnolipids (RL, Figure 1) are biosurfactants, that are produced by *Pseudomonas aeruginosa*, a Gram-negative rod-shaped bacterium [5–8]. RL are built up by one or two rhamnose sugar units as well as one to three β-hydroxy fatty acids, which can also be unsaturated. These highly functional biomolecules exhibit interesting biological and antibacterial properties, as described by Leisinger et al. [9]. Rhamnolipids possess some advantages, that make them interesting candidates as cleaning agents or additives for care applications in the consumer goods industry. They can be produced in a fermentation process from renewable nontropic material [10–11]. RL are also environmentally friendly biosurfactants because they are 100% biodegradable and are more compatible with water organisms than traditional surfactants.

In solution, surfactants self-assemble into micelles with different shapes (spherical, cylindrical, double layer, etc.). In aqueous solution, the surfactant molecules are oriented with their hydrophobic groups towards the interior of the micelle and their hydrophilic groups point towards the surrounding water molecules. However, in nonpolar solvents, the structure of the micelle is similar but reversed, with the hydrophilic groups in the micelle center and the hydrophobic groups are oriented towards the solvent. One structure which can be formed by surfactants are so-called wormlike micelles, that are characterized by a high anisotropic structure. These micelles can, if the concentration and dynamics of the surfactant micelles is right, build viscosity since the micelles behave similar to a polymer and can be nicely studied by rheology [12–13]. The underlying principle of wormlike micelles, which alter the rheology of the solvent is the same as for the “normal” system (micelles in water). However, the formation of wormlike micelles in organic solvents (respectively the one-dimensional growth) often requires the use of the correct amount of a primer [14]. Lecithin is the most prominent example of molecules forming reverse wormlike micelles [15–17]. The rheological properties of many oils can be fine-tuned by worm-like micelles in a more convenient way as compared to polymers and also the sensorial properties (like stringiness) are more pleasant [18]. However, the quality of the lecithin needs to be very high so that reverse wormlike micelles can be formed [19] and the amount of the primer needs to be well balanced as was described by Shchipunov when studying a lecithin/water system [20]. Gemini (bola) surfactants are a class of amphiphiles that has recently attracted a lot of attention [21–25]. They are made of two (or more) head groups that are connected by a spacer, that can be rigid or of variable length. In the present contribution we report the formation of viscoelastic reverse wormlike micellar solutions of RL ester with nonpolar solvents without the addition of any primer. The phase behavior and rheological properties were studied depending on the concentration and alkyl chain length of the rhamnolipid ester and on the temperature.

## Results and Discussion

### Materials and synthesis of dirhamnolipid ester

**Scheme 1:**
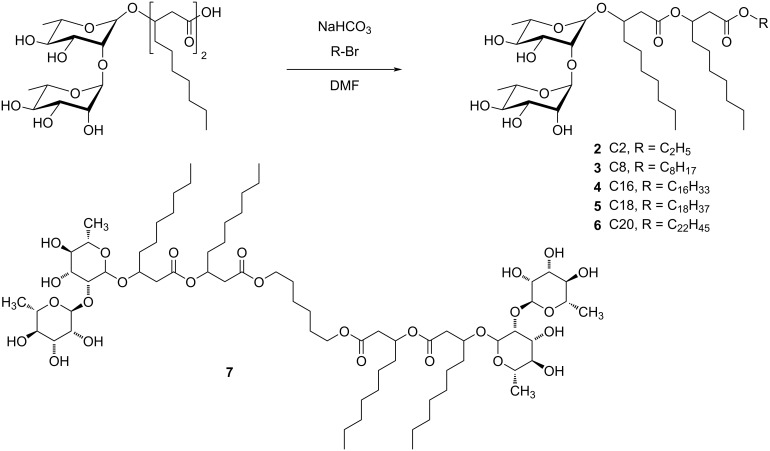
Synthesis of the dirhamnolipid esters and the chemical structure of **7**.

The used dirhamnolipid raw material stems from a biotechnological process. The purity is 90.8% with respect to the rhamnolipid acid form. Due to the biotechnological production, the alkyl chain length in the lipid part varies from C8 to C12, with a little portion of double bonds (according to HPLC–MS measurements this portion amounts to ca. 4–12%). A novel synthetic route was chosen for the synthesis of the dirhamnolipid esters. In contrast to the previously used methods [26–28], our approach is applicable for any desired alkyl chain length without the production of side products (Scheme 1). Under mild and basic conditions, with this S_N_2 reaction of the dirhamnolipid and any primary alkyl halide, the desired product was obtained with a high yield ranging from 70–85%. Also, a gemini-like structure **7** was synthesized.

### Solubility of the dirhamnolipid esters in various solvents

**Figure 2:**
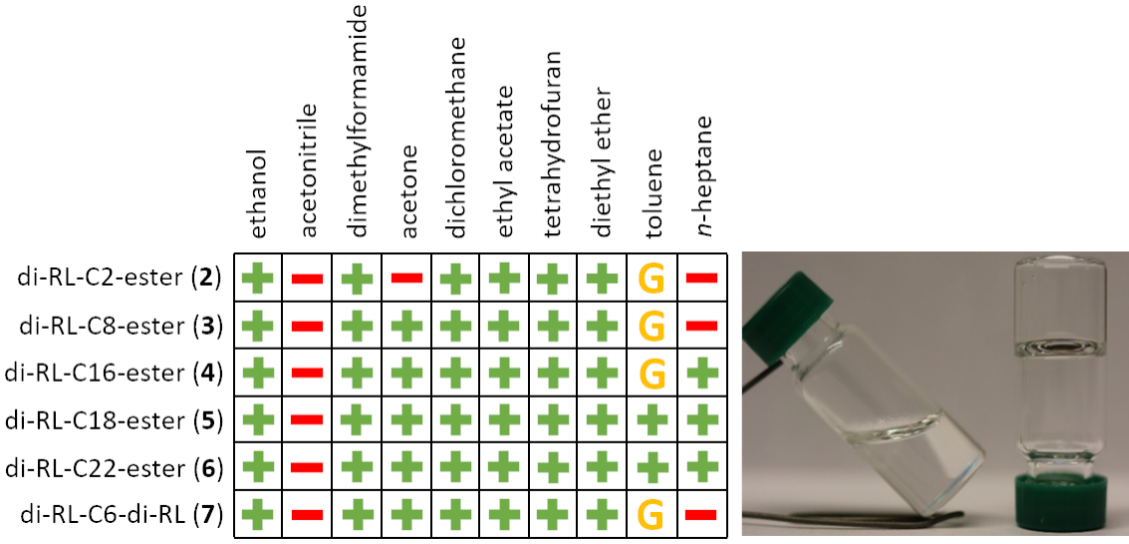
Solubility of the dirhamnolipid esters in various solvents (+ = soluble, − = insoluble, G = gel).

First, the solubility of the dirhamnolipid esters was investigated in various solvents (75 mg/mL) with respect to the alkyl chain length. The results are summarized in a solubility chart depicted in Figure 2. Whereas all dirhamnolipid esters are insoluble in acetonitrile, they are soluble in almost every medium polar solvent. In *n*-heptane, a nonpolar solvent, the shorter alkyl chain length dirhamnolipid esters and the bola derivative are insoluble, but with increasing chain length, the dirhamnolipid esters become soluble because of the decreasing polarity. An unusual behavior was observed when the dirhamnolipid esters were added to toluene. The more polar short chain length dirhamnolipid esters and **7** form a gel-like texture in toluene, indicating the formation of a network structure of the surfactant molecules. With increasing chain length of the dirhamnolipid esters, the ability to gel toluene vanishes.

### Rheological data

#### Temperature sweep

With this interesting observation we went on and performed rheological measurements to determine the viscoelastic properties of the dirhamnolipid ester/toluene gels. First, we performed temperature sweep measurements for the samples with dirhamnolipid esters of different alkyl chain length and the bola structure. Figure S1a in Supporting Information File 1 represent the plots for the change in the elastic (storage) modulus *G'* and the viscous (loss) modulus *G''* with respect to the temperature while heating. The overall qualitative behavior of the moduli is almost identical for the different samples. At a low temperature, *G'* was superior than *G''*, which indicates a solid-like behavior. With elevating temperature, *G'* drops significantly, while *G''* first rises and then also drops sharply. The course of the moduli can be explained with an inhomogeneous breakup of the three-dimensional structure. In the beginning, the network collapses into big chunks, while the surrounding material is still rigid. A further increase in the temperature leads to a dominating viscous behavior until the whole sample becomes fluid (*G''* > *G'*). Fluids are unable to store mechanical stress. Thus, the storage modulus drops to almost zero. For the cooling process, the change of the moduli mirrors the ones of the heating process (Figure S1b in Supporting Information File 1). We performed concentration-dependent temperature sweeps with dirhamnolipid ethyl ester **2** as it is the substance with the highest dynamic moduli. As expected, the moduli increase with an increasing concentration of the sample, while the qualitative pattern of the curves remains unchanged (Figure S2, Supporting Information File 1). Figure S3 (Supporting Information File 1) shows the thermal reversibility of the gel formation process. The phase transition temperature can be obtained from the temperature sweeps and is defined as the temperature where the dynamic moduli intercept. In an alternative representation, the phase transition temperature can also be described as the temperature where the phase angle equals δ = 45°. According to Equation 1, the phase angle is defined as:

[1] $$\tan\text{δ}=\frac{G''}{G'}$$

**Figure 3:**
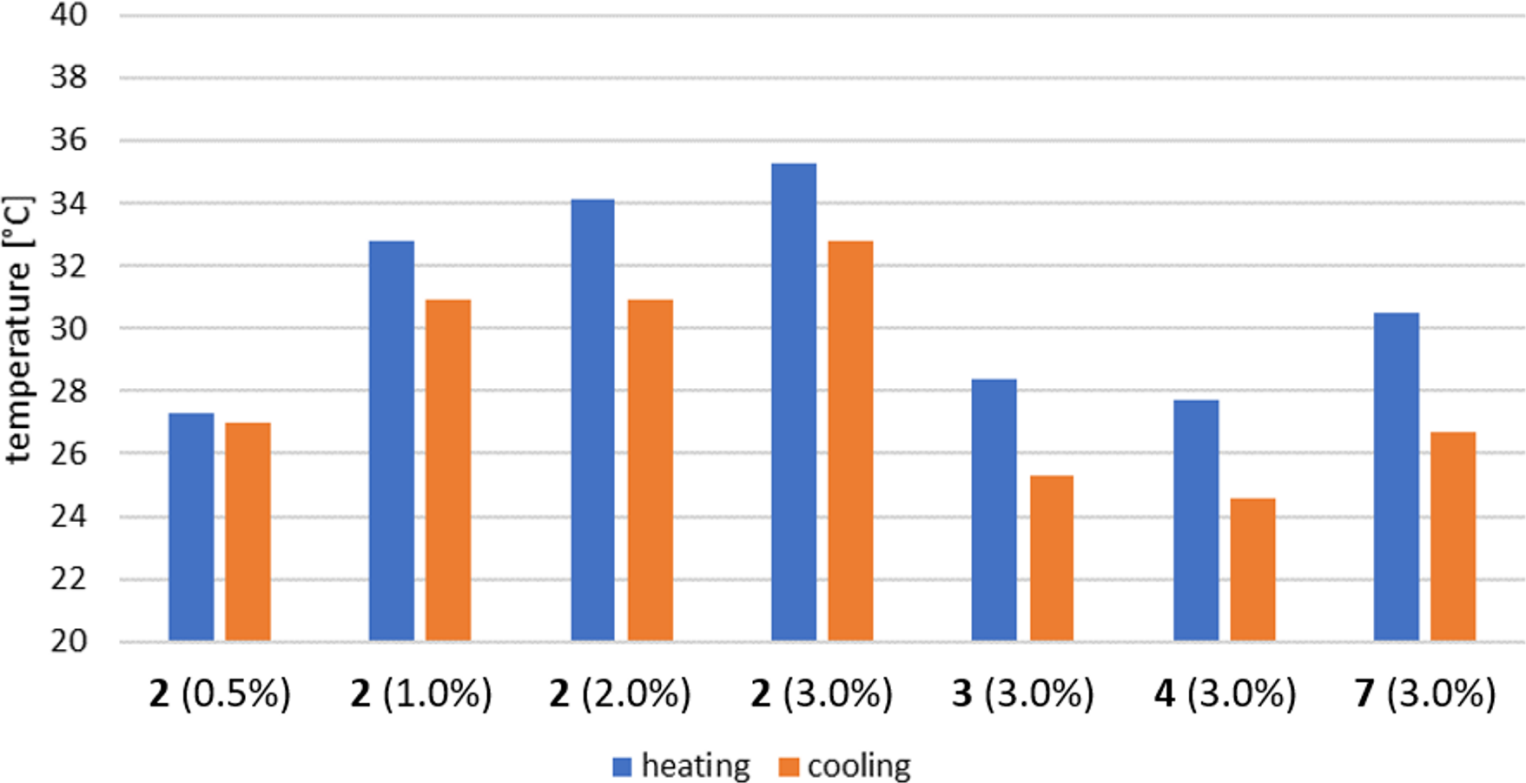
Phase transition temperature for the dirhamnolipid esters in toluene while heating (*T*_GS_, blue) and cooling (*T*_SG_, orange). The values were obtained from the phase angle (δ = 45°).

The results of the phase transition temperatures obtained from the phase angles are summarized in Figure 3. The highest transition temperatures were found for the dirhamnolipid ester with the shortest alkyl chain length. Also, the transition temperature rises with increasing concentration. With an increasing number of dirhamnolipid ester molecules a denser network is formed, which is confirmed by the higher values in *G'* and *G''* for higher concentrations. It should be emphasized that the formation of a gel structure was observed for concentrations as low as 0.5 wt %. Organogelators that are able to gel solvents at concentrations at <1% are classified as supergelators [29–31]. Furthermore, the results from the solubility tests where the ability of gelation decreases with an increasing alkyl chain length of the dirhamnolipid ester, could be confirmed. The transition temperature for the dirhamnolipid octyl ester **3** is approximately 7 °C lower than for the ethyl ester **2**, whereas the difference between the hexadecyl esters **4** and **3** is not as significant. The gemini-like structure **7** has transition temperatures that are between the ones of **2** and **3**. Different transition temperatures were found for the heating and cooling process, with *T*_GS_ > *T*_SG_. This hysteresis amounts to Δ*T* = 2–3 °C for the different samples. The lower transition temperatures from solution to gel can be caused by a slow formation of the three-dimensional network. The hysteresis gets smaller at low concentrations, indicating the approach of the critical gel-formation concentration.

#### Deformation sweep

**Figure 4:**
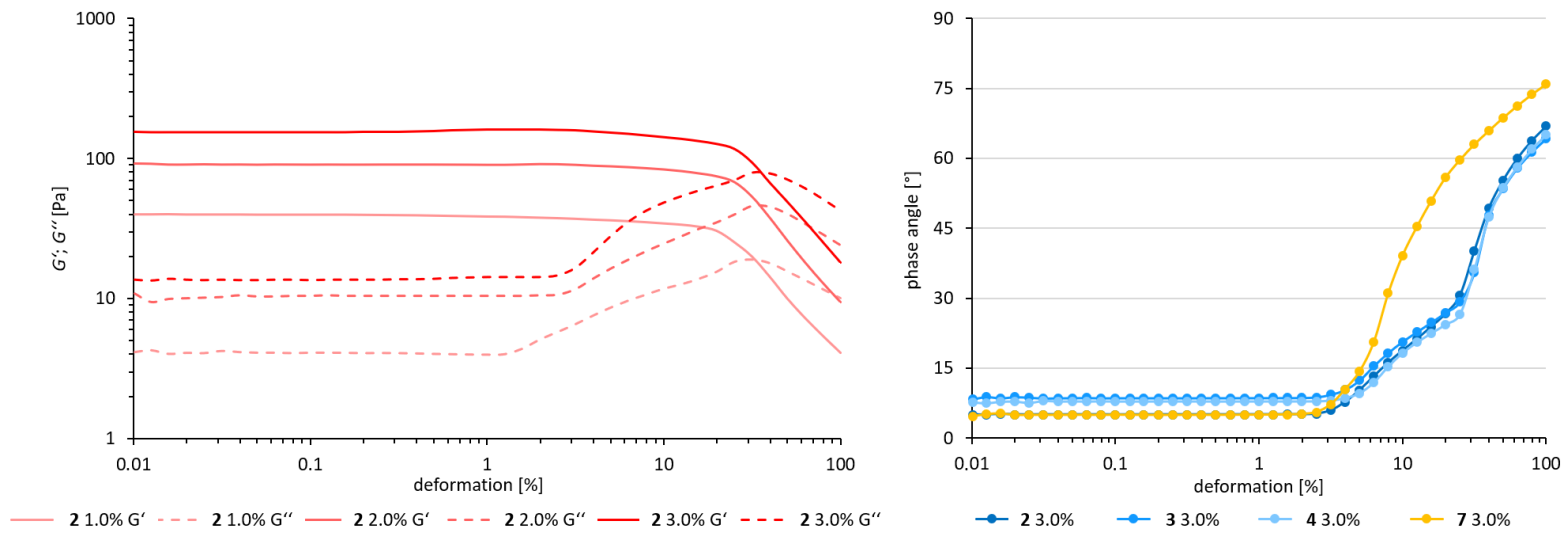
Amplitude sweep: double logarithmic plot of the dynamic moduli against the amplitude (deformation) for the dirhamnolipid ethyl ester with different concentrations in toluene (left), and a plot of the phase angle against the amplitude for the different dirhamnolipid esters (right) at 15 °C and a frequency of 1 rad/s.

To gather some more information about the viscoelastic material, a deformation sweep was performed. On the left side of Figure 4 the amplitude (deformation) is plotted against the dynamic moduli for **2** at different concentrations. In the linear viscoelastic (LVE) region at low deformation, the moduli stay constant, which means that the structure of the sample is still unchanged. At about γ = 2%, *G''* rises sharply, while *G'* remains almost flat until γ = 20%. This indicates that only a few interactions are released, suggesting the breakup of the network structure into big chunks. The surrounding material is still dominated by the storage modulus (*G'* > *G''*). Therefore, the sample still can be described as a viscoelastic solid. Both moduli intercept at γ = 35%, which marks the point where the sample turns from a viscoelastic solid to a viscous fluid. In a representation where the phase angle is plotted against the amplitude (Figure 4, right), it becomes apparent that the deformation processes are similar for the dirhamnolipid esters with a different alkyl chain length, whereas a different mechanism leads to the destruction of the network-like structure for the gemini surfactant **7**. For **7**, the transition from the solid to the liquid state occurs at much lower deformation of γ = 12.6% (δ = 45°).

#### Frequency sweep

**Figure 5:**
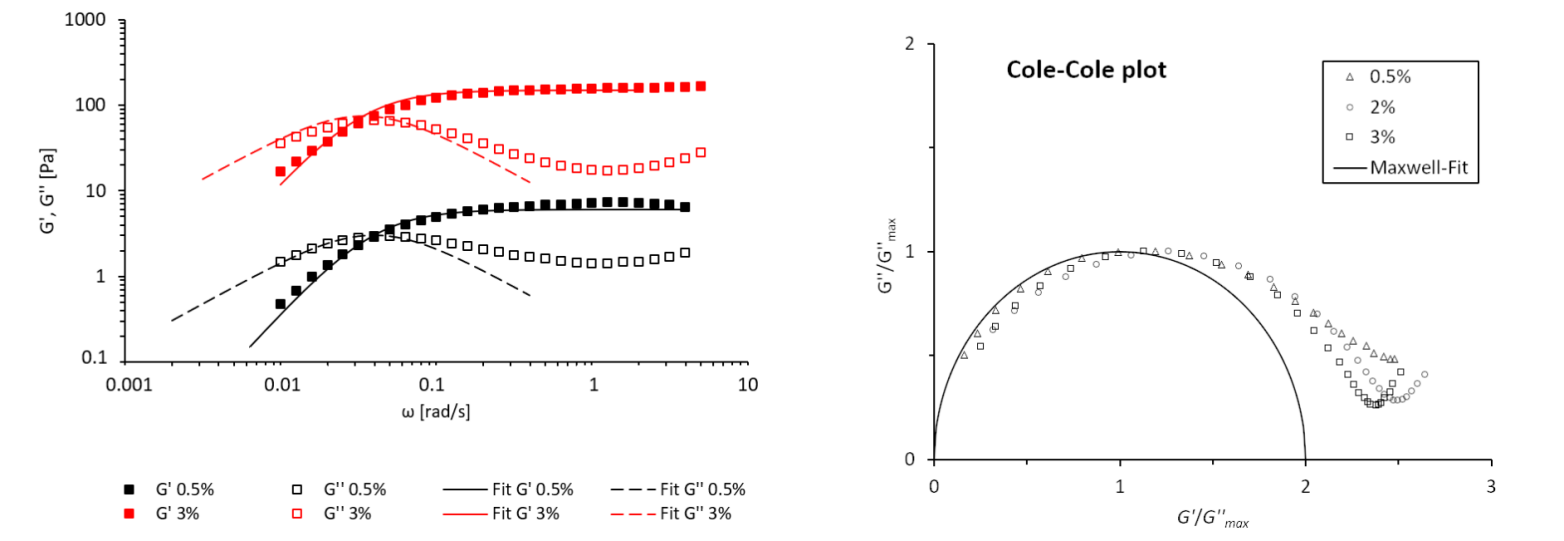
Frequency sweep: double logarithmic plot of the dynamic moduli against the frequency for the dirhamnolipid ethyl ester **2** at different concentrations in toluene at 15 °C and an amplitude of 0.2% (left), and the Cole–Cole plot for different concentrations as well as the Maxwell fit.

To investigate the viscoelastic properties in more detail, oscillatory-shear experiments were performed for the dirhamnolipid ethyl ester/toluene system. For the frequency sweep, the shear frequency ω was varied at a constant amplitude in the LVE region (γ = 0.2%) at 15 °C. The arrangement of the data for the dynamic moduli with respect to the shear frequency is shown in Figure 5.

In the low-frequency region, the material behaves liquid-like as *G''* is superior than *G'*. A solid behavior (*G'* > *G''*) was observed in the high-frequency region. Such a crossover of *G'* and *G''* is a characteristic of wormlike micelle (WLM) systems [12]. The viscoelastic behavior matches the Maxwell model for WLM very well. The course of the *G'* and *G''* curves can generally be fitted by Equation 2 and Equation 3 with a single relaxation time τ_R_ [32]:

[2]
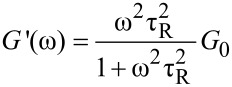


[3]
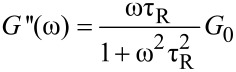


where the plateau molulus *G*_0_ corresponds to the fixed value of *G'* at a high ω, and the relaxation time τ*_R_* is defined as the reciprocal of the frequency where *G'* and *G''* intercept (ω_C_, Equation 4).

[4] $$\tau_{\text{R}}=\frac{1}{{\text{ω}}_{\text{C}}}$$

The mathematical fits of the values for the dynamic moduli (Figure 5) equal the experimental data of *G'* and *G''* in the low-ω region. This viscoelastic behavior is an evidence for the existence of reverse wormlike micelles (RWLM). The deviation from the Maxwell model in the high-ω region where the *G''* curve rises with an increase in ω even after reaching a minimum is considered to be due to the transition of the relaxation mode from the slower to the faster mode such as the Rouse-like motion for cylindrical micelles [33–34]. Another representation is the so-called Cole–Cole plot [35], where the normalized value for the loss modulus (*G''*/*G''*_max_) is plotted against the storage modulus (*G'*/*G''*_max_), is depicted in Figure 5. The semicircular arrangement centered at *G'*/*G''*_max_ is the result of the Maxwell-like behavior of the system. The deviation from the semicircle can be explained by the variation in the micelle length, and therefore varying relaxation times. For high *G'*, the upturn of the curves is due to the transition from the Maxwell model to a Rouse-like motion. The minimum of the storage modulus (*G''_min_*) is characteristic for WLM and is related to the contour length (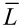) and the entanglement length (*l*_e_) by Equation 5 [36]:

[5] $$\frac{G_{0}}{G'{'}_{\min}}\approx \frac{\bar{L}}{l_{\text{e}}}$$

where the ratio 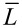/*l*_e_ represents the degree of entanglement. For the different concentrations, the values for the ratios are 4.2 (0.5%), 7.1 (2%), and 8.6 (3%), respectively. The number of entanglement points per micelle grows with increasing concentrations, thus building a tighter network. With the characteristic values of *l*_e_ (80–150 nm) for WLM [25], an average micelle length of 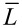 = 0.3–0.7 µm can be estimated. Another information can be obtained from *G''*_min_. The reciprocal of the frequency where G'' has a minimum approximately corresponds to the breaking/recombination time τ_b_ of the reverse micelles, which are self-organized systems that undergo constant scission and recombination processes in a dynamic equilibrium [36]. Therefore, τ_b_ is the mean lifetime of a chain before it breaks into two pieces. As expected, the scission time is independent from the concentration and amounts to τ_b_ = 0.13 s. With the knowledge of τ_R_ and τ_b_, the reptation time τ_rep_ can be estimated from Equation 6 [32]:

[6] $$\tau_{\text{R}}\approx \sqrt{\tau_{\text{b}}\cdot \tau_{\text{rep}}}$$

**Figure 6:**
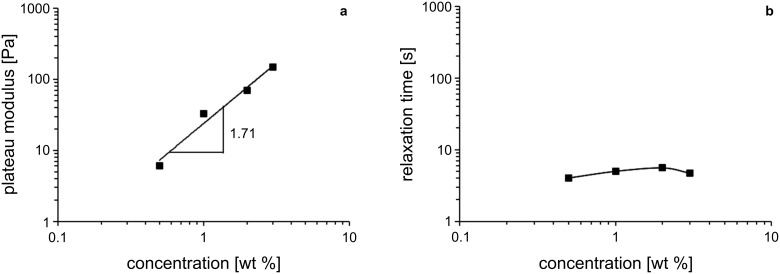
Double logarithmic plot of (a) the plateau modulus *G*_0_ and (b) the relaxation time *τ*_R_ against the concentration of **2** in toluene at 15 °C.

The reptation time is the reflection of the time that passes by while a micelle migrates one time along its contour length 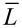. The value for τ_rep_ varies between 123–241 s, depending on the concentration (Table 1). A Maxwell model behavior is generally observed for τ_b_ << τ_rep_. This holds true for the experimental data obtained from the rheological measurements. The plateau molulus *G*_0_ is primarily related to the mesh size ξ of the entangled network by the relationship shown in Equation 7 [32]:

[7] $$G_{0}=\frac{k_{\text{B}}\cdot T}{\xi^{3}}$$

with *k*_B_ being the Boltzmann constant and *T* the temperature. The mesh size decreases with increasing concentration (Table 1). With an increasing number of micelles, the network becomes tighter so that ξ gets smaller. These results are in good agreement with the increasing number of entanglement points with increasing concentration. The relationship between the plateau modulus *G*_0_ as well as the relaxation time τ_R_ and the surfactant concentration is depicted in a double logarithmic plot in Figure 6. *G*_0_ is a measure for the number of micelles in the system whereas τ_R_ reflects the length of the inverse wormlike micelle [32]. As expected, the number of micelles rises with concentration as *G*_0_ gradually increases (Figure 6a). The exponent, which describes the relationship between *G*_0_ and the concentration, equals 1.71. This value is smaller than the one that is predicted for linear wormlike micelles by theoretical considerations (≈2.25) [32] but matches the values, that were found for branched wormlike micelles (≈1.8) [37–38]. Interestingly, τ_R_ is almost constant in the observed concentration range (Figure 6b). As τ_R_ reflects the length of the micelle, an increasing surfactant concentration does not lead to a one-dimensional growth of the micelles. This means that the increase in *G*_0_ is solely caused by the increasing number of micelles in the system, which then leads to a higher degree of entanglement. This phenomenon was previously observed in other surfactant systems with branched wormlike micelles [39–40]. The potential existence of intramicellar junctions for wormlike micelles was first proposed by Porte et al. [41]. This assumption was then supported by rheological measurements [42] and finally validated by transmission electron microscopy at cryogenic temperature (cryo-TEM) [43].

**Table 1:** Characteristic rheological values of reverse wormlike micelles from the dirhamnolipid ethyl ester **2**/toluene system.

conc. [wt % ]	*G*_0_ [Pa]	τ_R_ [s]	*G*''_min_ [Pa]	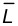 /*l*_e_	τ_rep_ [s]	ξ [nm]

0.5	6.1	4.0	1.43	4.2	123	86.7
1.0	33	5.0	3.41	9.7	192	49.4
2.0	70	5.6	9.85	7.1	241	38.4
3.0	148	4.7	17.30	8.6	170	30.0

#### Temperature-dependent rheology

**Figure 7:**
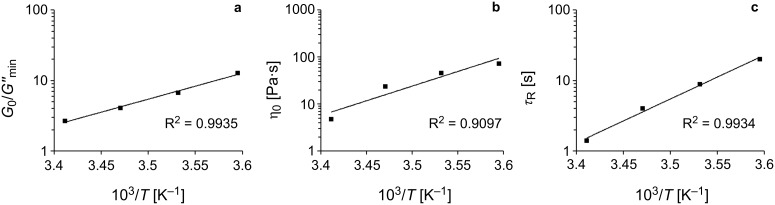
Semilogarithmic plot of (a) *G*_0_/*G''*_min_, (b) η_0_, and (c) τ_R_ against the inverse absolute temperature for the **2**/toluene system (0.5 wt %). The solid lines are fits to an Arrhenius-like behavior.

Next, the temperature dependence of the rheology for the RWLM will be described. Oscillatory-shear experiments were performed for the dirhamnolipid ethyl ester **2**/toluene (0.5 wt %) at temperatures varying from 5–20 °C. The results of the measurements are summarized in Table 2. Due to the low surfactant concentration, *G'* does not exhibit a well-definded plateau at high frequencies. Therefore, *G*_0_ was estimated from *G'*(ω_C_) through *G*_0_ = 2*G'*(ω_C_) for quasi-Maxwellian systems in the lowest limit of the surfactant concentration range [44]. The plateau moduli behave unusual as *G*_0_ first increases with increasing temperature and then decreases at the highest temperature. As described earlier, *G*_0_ represents the number of micelles in the system. The increase in the number of micelles can be explained by the breakup of big branched micelles into plenty of small ones. At elevated temperatures, the micelles can dissolve into surfactant monomers (dynamic equilibrium), which then leads to a decrease in the number of micelles. The relaxation time (τ_R_) gradually declines with increasing temperatures, indicating a reduction in the length of the micelles. Consequently, the shortened micelle lengths lead to a low-viscosity material at higher temperatures. The variations of *G''*_mi_*_n_*/*G*_0_, η_0_, and τ_R_ depending on the temperature are shown in Figure 7 as an Arrhenius plot (i.e., a semilogarithmic plot of the quantities versus 1/*T*). The values for all parameters fall on a straight line, indicating an exponential decrease.

**Table 2:** Rheological values obtained from the oscillatory-shear experiments for the dirhamnolipid ethyl ester **2**/toluene (0.5 wt %) system at different temperatures.

*T* [°C]	*G*_0_ [Pa]	τ_R_ [s]	η_0_ [Pa·s]	*G''*_min_ [Pa]	*G''*_min_/*G*_0_

5	3.60	20.1	72.4	0.28	0.078
10	5.18	8.9	46.1	0.77	0.149
15	5.86	4.0	23.4	1.43	0.244
20	3.42	1.4	4.8	1.28	0.374

From the rheology data (Figure 7a), the scission energy (*E*_sciss_) of the wormlike micelle system can be calculated via Equation 8 [32]:

[8] $$\frac{\bar{L}}{l_{\text{e}}}\approx \frac{G_{0}}{G'{'}_{\min}}\sim \exp\left(\frac{E_{\text{sciss}}}{2\cdot k_{\text{B}}\cdot T}\right)$$

The scission energy describes the energy that is necessary for the formation of two new micellar end caps. From the slope of the semilogarithmic plot we obtain *E*_sciss_ = 141 kJ/mol, and similar values have been reported for other surfactants [45–47]. From the literature, it is also known that the micellar contour length decreases with increasing temperature according to the following Arrhenius equations, Equation 9 and Equation 10 [32]:

[9] $$\tau_{\text{R}}=A\cdot \exp\left(\frac{E_{\text{a}}}{R\cdot T}\right)$$

[10] $$\eta_{0}=G_{0}\cdot A\cdot \exp\left(\frac{E_{\text{a}}}{R\cdot T}\right)$$

where *E*_a_ is the activation energy, *R* is the gas constant, and *A* is the preexponential factor. The semilogarithmic plots of η_0_ and τ_R_ versus 1/*T* (Figure 7b and c) indicate an Arrhenius plot like behavior, and the activation energy calculated from the slopes of the two plots equals 119 kJ/mol, close to those found in other wormlike micelles [48–52]. According to Equation 10, *G*_0_ is independent of the temperature, which is not true in our case. This might be due to the intramicellar branches and/or the inverse character of the micelles, as Equation 9 and Equation 10 are defined for normal linear micelles. The behavior of *G*_0_ varying with the temperature for inverse micelles was also found by other authors [53–54].

#### Microscopy

**Figure 8:**
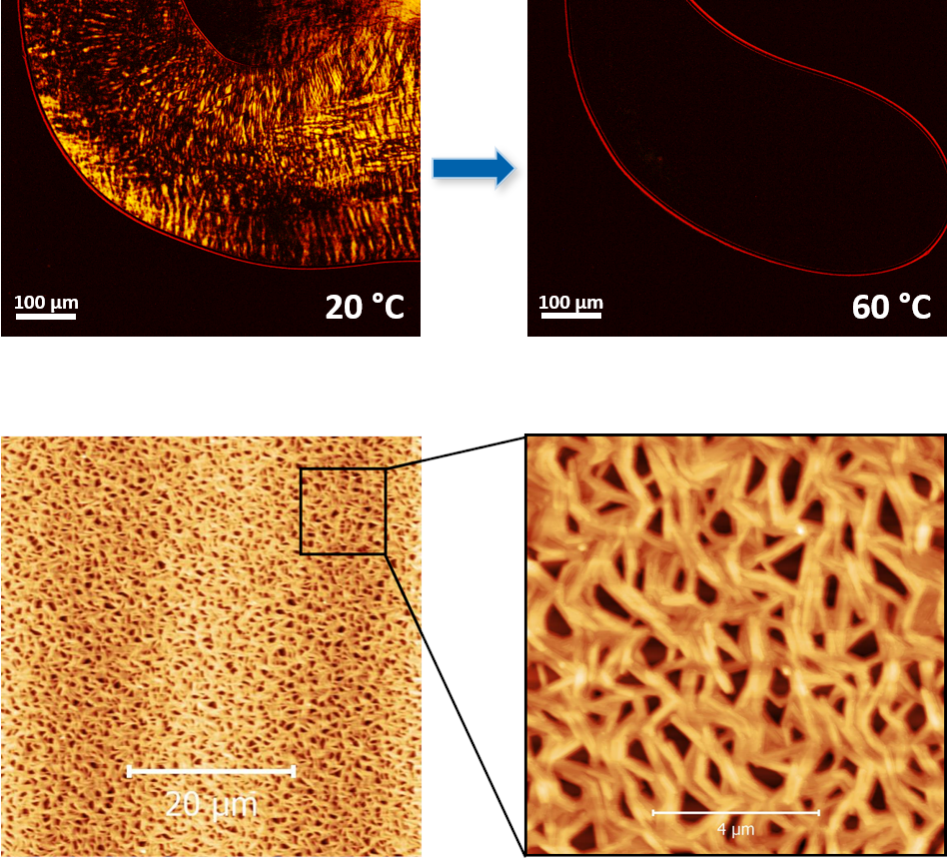
Polarized optical microscopy (POM) images of the **2**/toluene system (5 wt %) with crossed polarizers (10-fold magnification) at ambient temperature and after heating to 60 °C (upper panel), and atomic force microscopy (AFM) height images of **2**/toluene (50 µM, lower panel).

Polarized optical microscopy (POM) was applied to the gel-like material of the dirhamnolipid ethyl ester **2**/toluene (5 wt %) system. As shown in Figure 8, the gel-like sample is birefringent (optically anisotropic) at ambient temperature. The texture indicates a certain symmetry in the formed structure as the polarized light strongly interacts with the sample. When heating to 60 °C, the material becomes liquid and the order in the structure is lost. This leads to an optical isotropic medium, yielding only a dark background. Also, atomic force microscopy (AFM) height images were taken for the dirhamnolipid ethyl ester **2**/toluene system, as depicted in Figure 8. Here, a homogeneous network of entangled fibers was found. The heights of isolated fibers were determined (see Figure S4 in Supporting Information File 1) and diameters ranging from 20–35 nm were found.

#### Differential scanning calorimetry

Differential scanning calorimetry (DSC) analysis was performed to obtain some more information about the interactions that are responsible for the gel-like behavior. Five heating and cooling cycles were recorded for the gemini surfactant **7**/toluene system (5 wt %). The DSC curves (Figure S5 in Supporting Information File 1) once again demonstrate the thermal reversibility of the gelation process. There are peak maxima at the heating and cooling phases that can be assigned to the formation of the gel structure and its decomposition. For the heating phase, a maximum was found at 45 °C, the endothermic melting process of the sample. In the cooling phase, the exothermic formation of the network is indicated by a maximum at 42 °C. This hysteresis of Δ*T* = 3 °C is consistent with the results obtained from the rheological measurements. Furthermore, the melting and gel formation enthalpies were extracted from the DSC data (Table S2, Supporting Information File 1). Both values are identical and amount to Δ*H*_GS,SG_ = ±7.2 kJ/mol, which is in the order of strong dispersion energies. This further proves the formation of network-like structures by the entanglement of RWLM due to attractive dispersion interactions between the alkyl chains of neighboring micelles. At elevated temperatures these interactions are weakened. Thus, the RWLM can move freely within the solvent and the material becomes fluid.

## Conclusion

**Figure 9:**
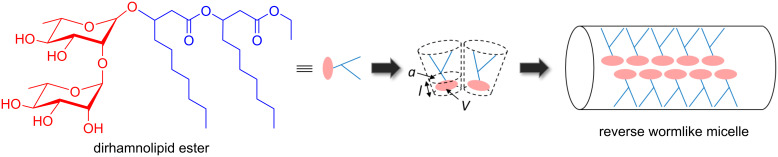
Schematic representation of the formation of RWLM by dirhamnolipid esters.

In summary, we could show that the gelation of nonpolar solvents by dirhamnolipid esters is caused by the formation of branched RWLM. These micelles are entangled at ambient temperature, so that a network-like structure is formed. An illustration of the formation of RWLM by the dirhamnolipid esters is shown in Figure 9. The hydrophilic sugar parts are oriented towards the interior of the micelle and the lipophilic alkyl chains are pointed towards the surrounding solvent molecules. The morphology of the micelles (spherical, cylindrical, vesicle, etc.) can be predicted by the packing parameter *P* of the surfactant [55–56]. The packing parameter *P* is defined by geometrical quantities as in Equation 11 [57]:

[11] $$P=\frac{V}{a\cdot l}$$

where, for reverse micelles, *V* is the volume of the hydrophilic part, *a* is the area per molecule at the aggregate interface, and *l* is the length of the hydrophilic part. For the dirhamnolipid esters the frustum-like geometry of the molecules is the reason why wormlike micelles are formed. The basis of the frustum is widened the longer the alkyl chain is, which leads to the formation of more spherical micelles, resulting in a loosener network. This also explains the results of the phase transition temperature, where lower values were found for longer alkyl chain lengths. Often, the formation of WLM is achieved by an addition of a third compound (primer), which induces the transition from spherical micelles to WLM. The packing parameter of the surfactant can be tuned systematically, e.g., by changing the size of the head group [58]. For reverse micelles, primers are polar substances that position themselves between neighboring surfactant head groups, and thus widening their distance. The packing parameter is manipulated in such a way that the formation of wormlike micelles occurs. A review of Palazzo showcases a lot of examples, where RWLM are formed only after the addition of a primer (ternary systems) [14]. Our system is a very rare case where no primer is needed for the formation of RWLM. To our knowledge, there is only one other example of a binary system with such a behavior [59]. Moreover, the surfactant, that was used as a precursor exhibits a certain distribution of the molecular weight but still was able to form reverse wormlike micelles, whereas most surfactants need to be purified, e.g., by recrystallization [54]. Also, the prototype of reverse micelles, organogels by lecithin, has high expectations towards the purity of the molecules [19].

## Experimental

### Differential scanning calorimetry

DSC data were obtained using a Perkin Elmer STA 6000 with a heating/cooling rate of 10 K/min (sample weight ≈ 2 mg).

### Polarized optical microscopy

Polarized optical microscopy (POM) images were taken on an Olympus BX41 microscope equipped with crossed polarizers, a hot stage, and an OptixCam Summit KZ OCS-SK2-52X microscope camera.

### Atomic force microscopy

Atomic force microscopy (AFM) images were performed in the tapping mode using a NanoDrive Controller with an Innova Scanning Probe Microscope (Veeco) and an N-type silicon cantilever (Olympus AC 16TS). The samples were prepared by drop-coating the solution (50 µM) on a freshly cleaned mica surface (Plano) and waiting for 12 h. The AFM data were analyzed using the Gwyddion-2.53 software.

### Rheological measurements

The rheological data were obtained using an Anton Paar MCR 301 rheometer equipped with a cylindrical geometry (CC27 – measuring unit). The temperature was controlled and adjusted with a Peltier element.

## Supporting Information

File 1 Synthesis of the compounds and additional Figures and Tables (viscosity).
