# Supplementary material for: Dirhamnolipid ester – formation of reverse wormlike micelles in a binary (primerless) system
Source: Beilstein J Org Chem. 2020 Nov 19;16:2820–30. doi: 10.3762/bjoc.16.232 (PMC7684688; doi:10.3762/bjoc.16.232)
Supplement: File 1 — Synthesis of the compounds and additional Figures and Tables (viscosity). [file Beilstein_J_Org_Chem-16-2820-s001.pdf]

BEILSTEIN JOURNAL OF ORGANIC CHEMISTRY

Supporting Information

for

Dirhamnolipid ester – formation of reverse wormlike micelles in a binary (primerless) system

David Liese, Hans Henning Wenk, Xin Lu, Jochen Kleinen and Gebhard Haberhauer

Beilstein J. Org. Chem. **2020,** *16,* 2820–2830. doi:10.3762/bjoc.16.232

Synthesis of the compounds and additional Figures and Tables (viscosity)

License and Terms: This is a supporting information file under the terms of the Creative Commons Attribution License (https://creativecommons.org/licenses/by/4.0). Please note that the reuse, redistribution and reproduction in particular requires that the authors and source are credited.
The license is subject to the *Beilstein Journal of Organic Chemistry* terms and conditions: (https://www.beilstein-journals.org/bjoc)

Table of contents

1. Synthesis of new compounds S2

2. Figures and tables S11

3. Viscosity S15

4. ^{1}H NMR and ^{13}C NMR spectra of the new compounds S17

5. References S23

1. Synthesis of the new compounds

General remarks: All chemicals were reagent grade and were used as purchased from ABCR, Alfa Aesar, Acros Organics, Carbolution, TCI, or Sigma-Aldrich. Reactions were monitored by TLC analysis with silica gel 60 F_{254} thin-layer plates with $KMnO_4$ stain solution. Flash chromatography was carried out on silica 60 (40–63 µm, 230–400 mesh). ^{1}H and ^{13}C NMR spectra were measured with Avance NEO 400, DMX 300, and Avance HD 600 spectrometers, respectively. All chemical shifts (δ) are given in ppm. The spectra were referenced to the peak for the protium impurity in the deuterated solvents indicated in brackets in the analytical data ($CDCl_3$, ^{1}H: 7.26 ppm, ^{13}C: 77.16 ppm). Signal multiplicities for ^{1}H NMR were determined as s (singlet), t (triplet), q (quartet), bs (broad signal), and m (multiplet). ^{13}C NMR spectra were measured with ^{1}H decoupling. ^{1}H and ^{13}C assignment was achieved via HSQC, HMBC, and COSY spectra. The ^{13}C signals were referred to p (primary), s (secondary), t (tertiary), and q (quaternary) carbon atoms. IR absorption spectra were recorded with a Varian 3100 FT-IR spectrophotometer.

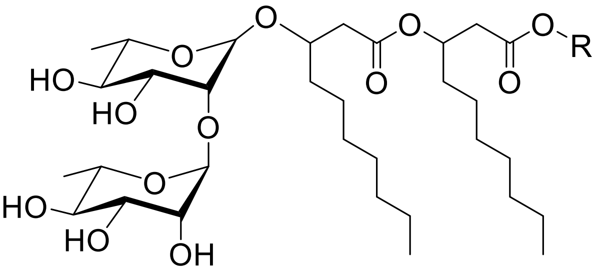

2 C2, R = C_2H_5
3 C8, R = C_8H_{17}
4 C16, R = $C_{16}H_{33}$
5 C18, R = $C_{18}H_{37}$
6 C20, R = $C_{22}H_{45}$

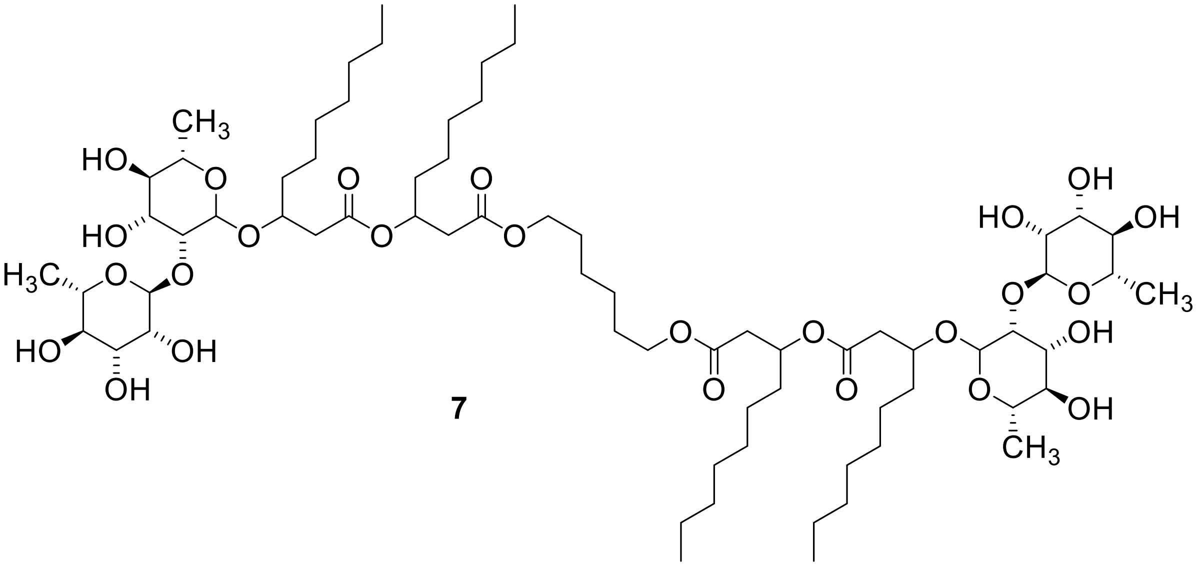

7

Synthesis of the dirhamnolipid esters 2–7. The dirhamnolipid **1** (664 mg, ≈ 0.98 mmol, 1.0 equiv), *n*-alkyl bromide (0.98 mmol, 1.0 equiv), and sodium bicarbonate ($NaHCO_3$; 103 mg, 1.23 mmol, 1.25 equiv) were stirred in 5 mL dimethylformamide at 100 °C for one hour. The reaction mixture is treated with water and extracted four times with 20 mL diethyl ether. The combined organic phases were washed four times with aqueous lithium chlorid solution (5 wt %) and dried with sodium sulfate. The solvent was removed in vacuo and the residue was purified by flash chromatography (SiO_2, dichloromethane → dichloromethane/methanol 9:1) to yield the

desired product (0.55 mmol, 56%) as highly viscous fluid. R_f (dichloromethane/methanol 9:1) = 0.1.

Characterization of the dirhamnolipid ethyl ester 2

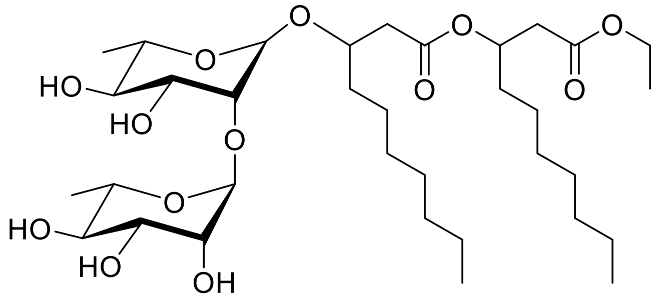

2

^{1}H-NMR (400 MHz, CDCl$_3$), *δ*[ppm]:

5.21 (m, 1 H), 4.93 (s, 1 H), 4.88 (s, 1 H), 4.25 (bs, 5 H, OH), 4.15 (m, 1 H), 4.12 (q, $^{3}J_{H,H}$ = 7.1 Hz, 2 H), 4.06 (m, 1 H), 3.77 (m, 4 H), 3.63 (m, 1 H), 3.49 (m, 1 H), 3.34 (m, 1 H), 2.53 (m, 4 H), 1.59 (m, 2 H), 1.50 (m, 2 H), 1.26 (m, 25 H), 1.25 (t, $^{3}J_{H,H}$ = 7.1 Hz, 3 H), 0.87 (m, 6 H).

^{13}C-NMR (101 MHz, CDCl$_3$), *δ*[ppm]:

171.13, 171.05, 102.73, 97.24, 79.77, 73.63, 72.65, 71.51, 71.42, 70.93, 70.74, 69.06, 68.68, 61.13, 40.20, 39.27, 34.12, 33.15, 31.95, 31.88, 31.64, 29.81, 29.62, 29.47, 29.33, 29.27, 25.21, 24.88, 22.76, 22.66, 22.60, 17.79, 14.28, 14.24, 14.22.

IR (ATR), $\tilde{\nu}$ [cm^{-1}]:

3366, 2924, 2857, 1736, 1456, 1377, 1302, 1260, 1161, 1126, 1028, 982, 916, 837, 808, 706.

Characterization of the dirhamnolipid octyl ester 3

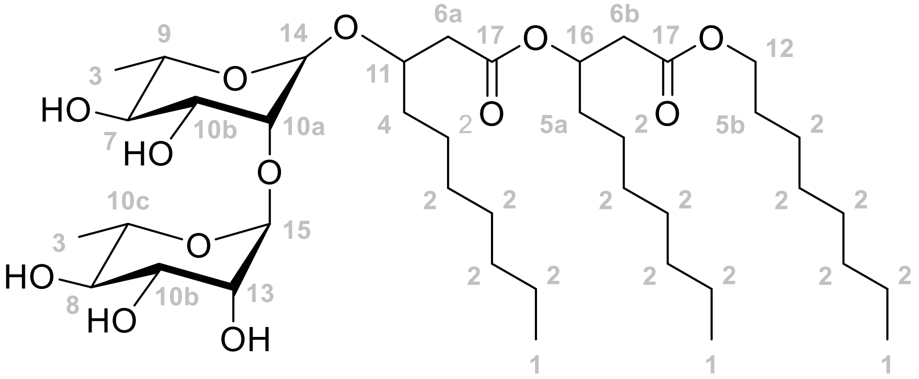

3

^{1}H-NMR (600 MHz, $CDCl_3$), *δ*[ppm]:

5.18 (m, 1 H, $C^{16}H$), 4.92 (s, 1 H, $C^{15}H$), 4.90 (s, 1 H, $C^{14}H$), 4.63 (bs, 5 H, OH), 4.18 (m, 1 H, $C^{13}H$), 4.05 (t, $^3J_{H,H}$ = 6.7 Hz, 2 H, $C^{12}H_2$), 4.05 (m, 1 H, $C^{11}H$), 3.77 (m, 4 H, $C^{10}H$), 3.63 (m, 1 H, C^9H), 3.50 (m, 1 H, C^8H), 3.38 (m, 1 H, C^7H), 2.53 (m, 4 H, C^6H_2), 1.61 (m, 4 H, C^5H_2), 1.50 (m, 2 H, C^4H_2), 1.26 (m, 38 H, C^2H_2, C^3H_3), 0.87 (m, 9 H, C^1H_3).

^{13}C-NMR (151 MHz, $CDCl_3$), *δ*[ppm]:

171.16 (q, C^{17}), 170.99 (q, C^{17}), 102.70 (t, C^{15}), 97.32 (t, C^{14}), 79.77 (t, C^{10a}), 73.79 (t, C^{11}), 73.16 (t, C^7), 72.63 (t, C^8), 71.44 (t, C^{10b}), 70.99 (t, C^{16}), 70.70 (t, C^{13}), 69.04 (t, C^{10c}), 68.69 (t, C^9), 65.32 (s, C^{12}), 40.25 (s, C^{6a}), 39.27 (s, C^{6b}), 34.06 (s, C^{5a}), 33.18 (s, C^4), 31.93 (s, C^2), 29.81 (s, C^2), 29.34 (s, C^2), 28.66 (s, C^{5b}), 26.09 (s, C^2), 25.23 (s, C^2), 24.87 (s, C^2), 22.76 (s, C^2), 17.77 (p, C^3), 17.68 (p, C^3), 14.23 (p, C^1).

IR (ATR), $\tilde{\nu}$ [cm^{-1}]:

3379, 2955, 2924, 2855, 1736, 1456, 1379, 1283, 1260, 1163, 1126, 1032, 982, 918, 837, 808, 721, 706.

Characterization of the dirhamnolipid hexadecyl ester 4

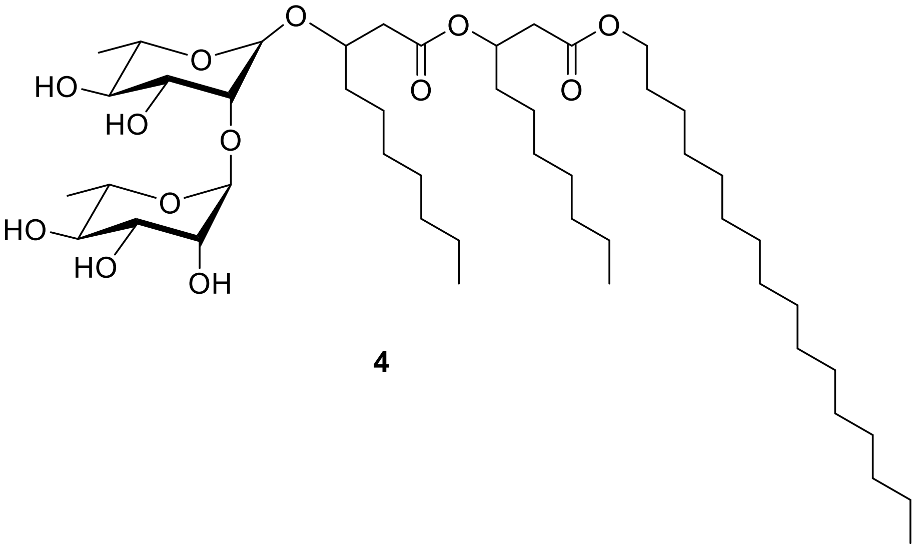

4

^{1}H-NMR (300 MHz, $CDCl_3$), *δ*[ppm]:

5.19 (m, 1 H), 4.93 (s, 1 H), 4.89 (s, 1 H), 4.33 (bs, 5 H, OH), 4.15 (s, 1 H), 4.05 (m, 3 H), 3.76 (m, 4 H), 3.62 (m, 1 H), 3.49 (m, 1 H), 3.34 (m, 1 H), 2.53 (m, 4 H), 1.60 (m, 4 H), 1.50 (m, 2 H), 1.25 (m, 52 H), 0.87 (m, 9 H).

^{13}C-NMR (151 MHz, $CDCl_3$), *δ*[ppm]:

171.17, 171.02, 102.70, 97.25, 79.74, 73.67, 73.22, 72.74, 71.52, 71.43, 71.00, 70.73, 69.03, 68.70, 65.34, 40.26, 39.27, 34.07, 33.15, 32.07, 31.97, 31.90, 29.87, 29.81, 29.80, 29.74, 29.51, 29.47, 29.35, 29.29, 28.68, 26.13, 25.24, 24.88, 22.83, 22.77, 17.79, 17.69, 14.26, 14.24, 14.22.

IR (ATR), $\tilde{\nu}$ [cm^{-1}]:

3366, 2922, 2853, 1736, 1456, 1379, 1283, 1163, 1128, 1045, 982, 916, 880, 837, 810, 721.

Characterization of the dirhamnolipid octadecyl ester 5

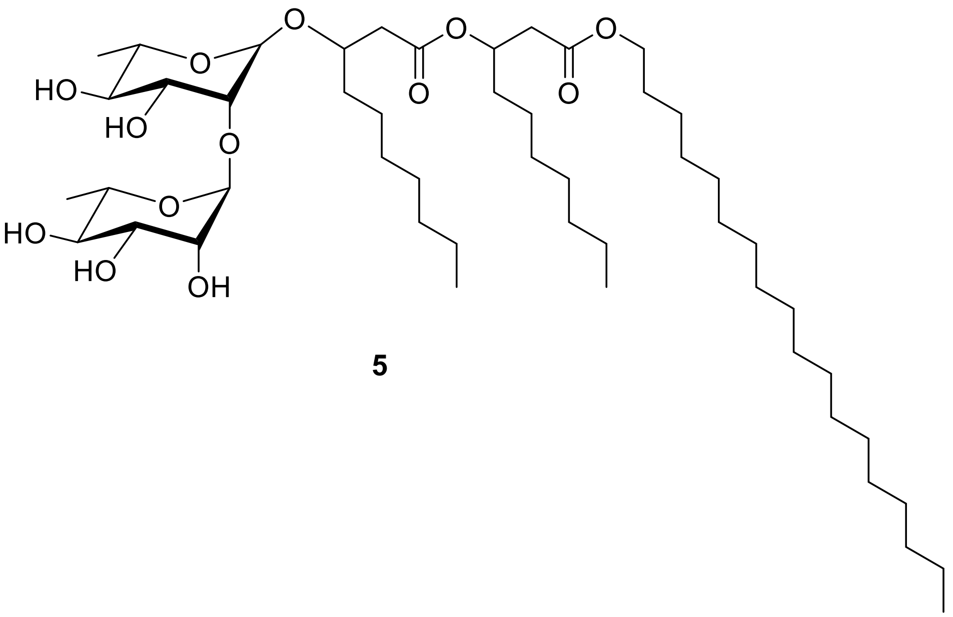

5

^{1}H-NMR (300 MHz, $CDCl_3$), δ[ppm]:

5.19 (m, 1 H), 4.93 (s, 1 H), 4.89 (s, 1 H), 4.32 (bs, 5 H, OH), 4.16 (s, 1 H), 4.05 (m, 3 H), 3.77 (m, 4 H), 3.63 (m, 1 H), 3.49 (m, 1 H), 3.34 (m, 1 H), 2.53 (m, 4 H), 1.60 (m, 4 H), 1.50 (m, 2 H), 1.25 (m, 55 H), 0.87 (m, 9 H).

^{13}C-NMR (151 MHz, $CDCl_3$), δ[ppm]:

171.17, 171.01, 102.71, 97.26, 79.75, 73.71, 73.20, 72.69, 71.52, 71.43, 70.99, 70.71, 69.03, 68.69, 65.34, 40.25, 39.27, 34.06, 33.16, 32.07, 31.97, 31.90, 29.87, 29.86, 29.81, 29.74, 29.51, 29.47, 28.68, 26.14, 25.24, 24.88, 22.83, 22.77, 17.78, 17.68, 14.26, 14.24, 14.22.

IR (ATR), $\tilde{\nu}$ [cm^{-1}]:

3379, 2922, 2853, 1738, 1456, 1377, 1283, 1260, 1163, 1126, 1036, 982, 916, 880, 837, 810, 721.

Characterization of the dirhamnolipid docosyl ester 6

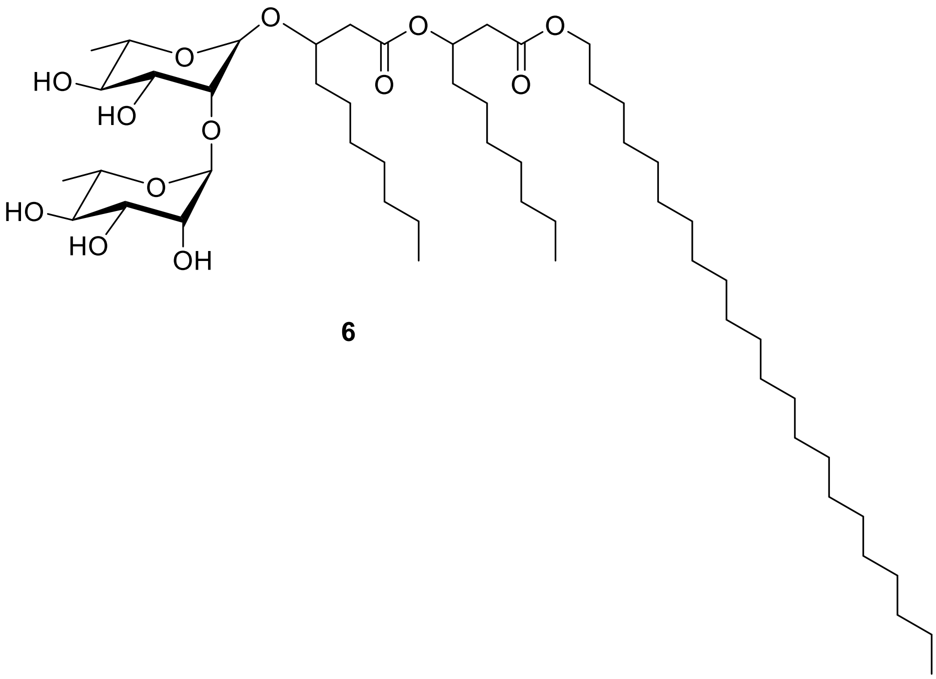

6

^{1}H-NMR (400 MHz, CDCl$_3$), δ[ppm]:

5.20 (m, 1 H), 4.95 (s, 1 H), 4.90 (s, 1 H), 4.14 (s, 1 H), 4.06 (m, 3 H), 3.78 (m, 4 H), 3.64 (m, 1 H), 3.49 (m, 1 H), 3.35 (m, 1 H), 3.16 (bs, 5 H, OH), 2.53 (m, 4 H), 1.61 (m, 4 H), 1.50 (m, 2 H), 1.25 (m, 64 H), 0.88 (m, 9 H).

^{13}C-NMR (151 MHz, CDCl$_3$), δ[ppm]:

171.26, 171.05, 102.59, 97.05, 79.67, 73.57, 73.38, 72.84, 71.49, 70.98, 70.74, 68.94, 68.65, 65.39, 63.26, 40.21, 39.27, 34.10, 33.09, 32.07, 31.97, 31.91, 29.89, 29.86, 29.81, 29.75, 29.51, 29.48, 29.36, 29.30, 28.68, 26.14, 25.24, 24.87, 22.84, 22.77, 17.74, 17.67, 14.27, 14.25, 14.23.

IR (ATR), $\tilde{\nu}$ [cm^{-1}]:

3379, 2916, 2851, 1738, 1466, 1377, 1310, 1161, 1130, 1030, 980, 916, 891, 839, 808, 719.

Characterization of the dirhamnolipid-C6-dirhamnolipid 7

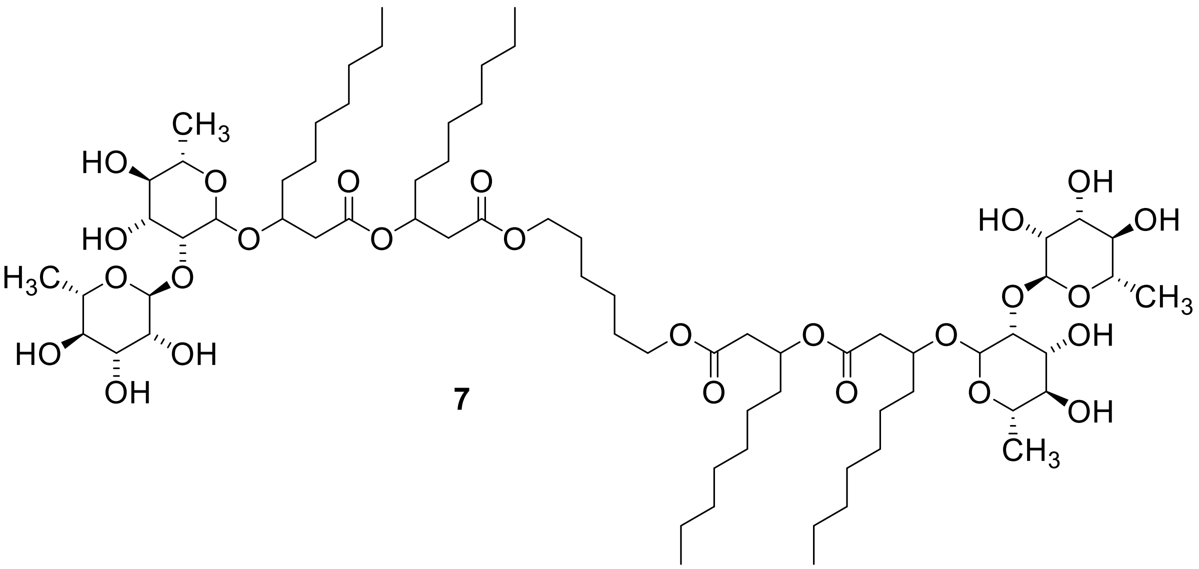

7

^{1}H-NMR (300 MHz, CDCl3), *δ*[ppm]:

5.21 (m, 2 H), 4.92 (s, 2 H), 4.90 (s, 2 H), 4.34 (bs, 10 H, OH), 4.19 (s, 2 H), 4.07 (m, 6 H), 3.76 (m, 8 H), 3.63 (m, 2 H), 3.50 (m, 2 H), 3.37 (m, 2 H), 2.56 (m, 8 H), 1.61 (m, 8 H), 1.50 (m, 4 H), 1.37 (m, 4 H), 1.26 (m, 51 H), 0.87 (m, 12 H).

^{13}C-NMR (151 MHz, CDCl3), *δ*[ppm]:

171.36, 171.13, 102.75, 96.93, 79.87, 73.36, 73.12, 72.52, 71.37, 70.87, 70.71, 69.07, 68.68, 65.12, 40.06, 39.27, 34.18, 33.09, 32.02, 31.95, 31.89, 31.68, 29.83, 29.63, 29.58, 29.50, 29.43, 29.34, 29.28, 28.47, 25.53, 25.24, 24.88, 22.81, 22.76, 22.66, 22.61, 17.77, 17.70, 14.25, 14.22.

IR (ATR), $\tilde{\nu}$ [cm^{-1}]:

3378, 2924, 2857, 1736, 1456, 1379, 1302, 1261, 1163, 1125, 1028, 980, 916, 880, 837, 808, 721, 704.

Table S1: Results of the HPLC-LRMS (ESI) analysis of the dirhamnolipid esters. (t_R = retention time, n.d. = not determinable, C12:1 corresponds to a β-hydroxy-*n*-dodecanoic acid rest containing a C–C double bond). Instrument: Agilent 6320 Ion Trap.

		t_R [min]	m/z	$[M+NH_4]^+$ [g/mol]
Di-RL-C2-ester	C8-C8	4.4	642.0	640.8
	C8-C10	6.6	669.2	668.8
	C10-C10	8.4	696.9	696.9
	C10-C12:1	9.6	723.6	722.9
	C10-C12	10.2	725.7	725.0
Di-RL-C8-ester	C8-C8	10.1	726.1	725.0
	C8-C10	11.7	753.2	753.0
	C10-C10	13.1	780.9	781.1
	C10-C12:1	13.9	807.9	807.1
	C10-C12	14.5	810.1	809.1
Di-RL-C16-ester	C8-C8	16.4	838.5	837.2
	C8-C10	17.6	865.2	865.2
	C10-C10	18.9	893.0	893.3
	C10-C12:1	19.6	919.4	919.3
	C10-C12	20.0	921.5	921.3
Di-RL-C18-ester	C8-C8	17.8	866.6	865.2
	C8-C10	19.1	893.1	893.3
	C10-C10	20.3	921.0	921.3
	C10-C12:1	20.9	947.4	947.4
	C10-C12	21.3	949.3	949.4
Di-RL-C22-ester	C8-C8	20.8	921.9	921.3
	C8-C10	21.8	949.1	949.4
	C10-C10	22.9	977.1	977.4
	C10-C12:1	n.d.	1003.3	1003.5
	C10-C12	23.7	1005.1	1005.5

2. Figures and tables

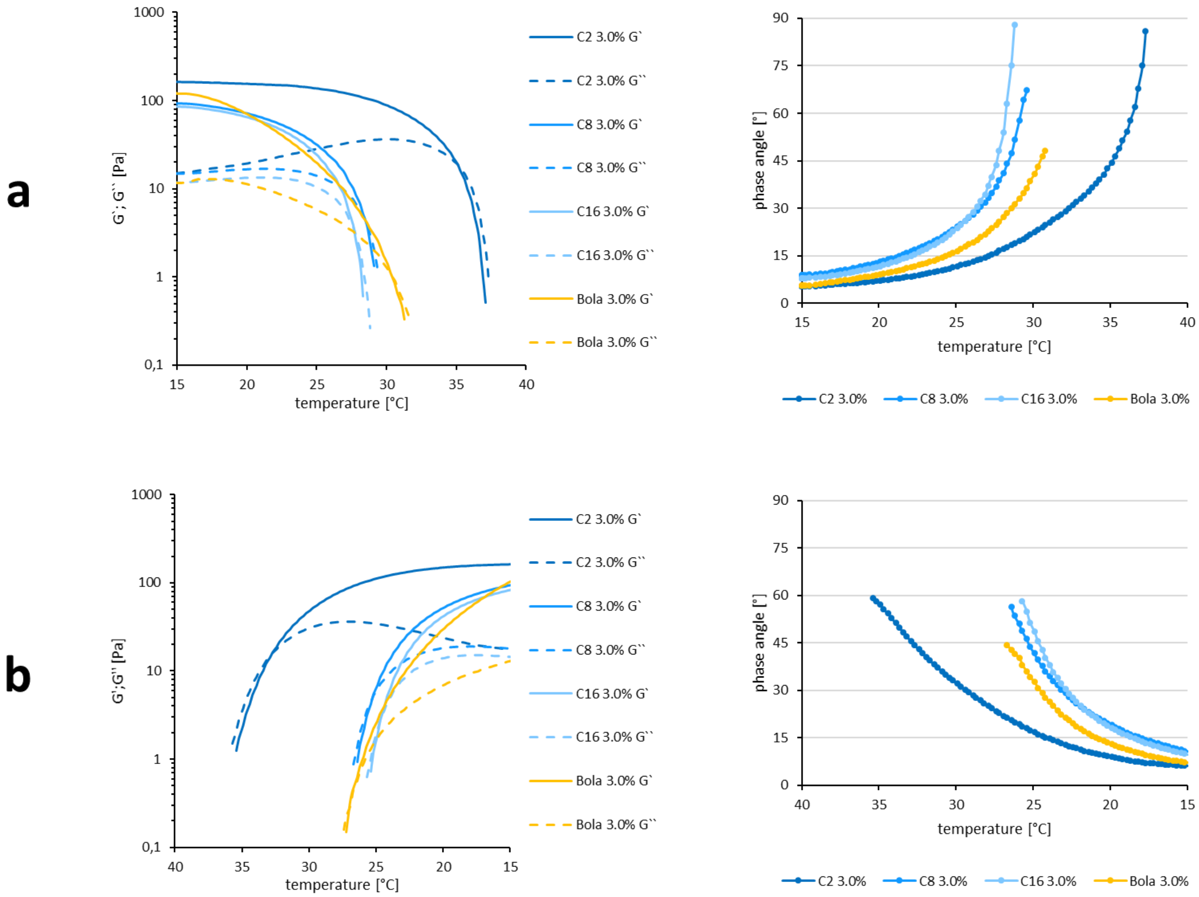

Figure S1: Variation of *G*’ and *G”* (left) and the phase angle (right) with respect to the temperature for the dirhamnolipid esters **2**, **3**, **4**, and **7** in toluene (3 wt %) while (a) heating and (b) cooling [*A* = 0.5%, *f* = 1 rad/s].

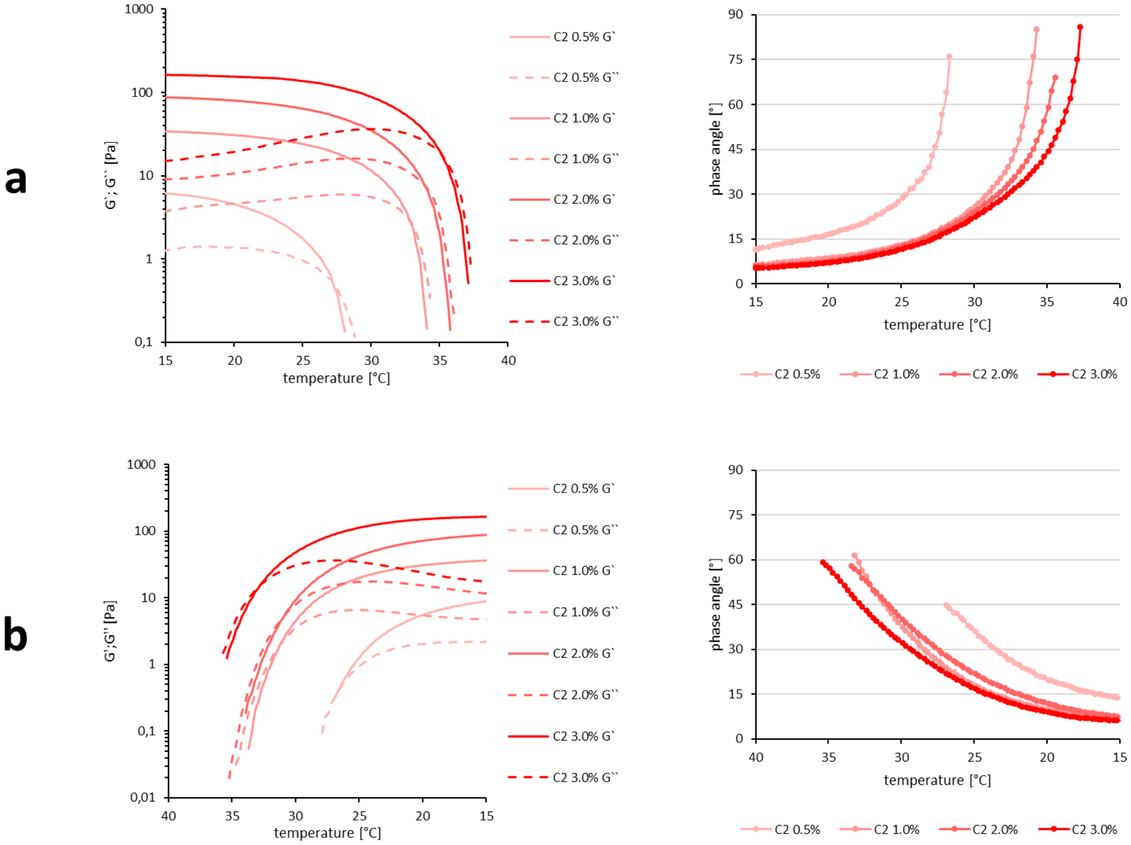

Figure S2: Variation of *G'* and *G''* (left) and the phase angle (right) with respect to the temperature for dirhamnolipid ethyl ester **2** at different concentrations in toluene while (a) heating and (b) cooling [*A* = 0.5%, *f* = 1 rad/s].

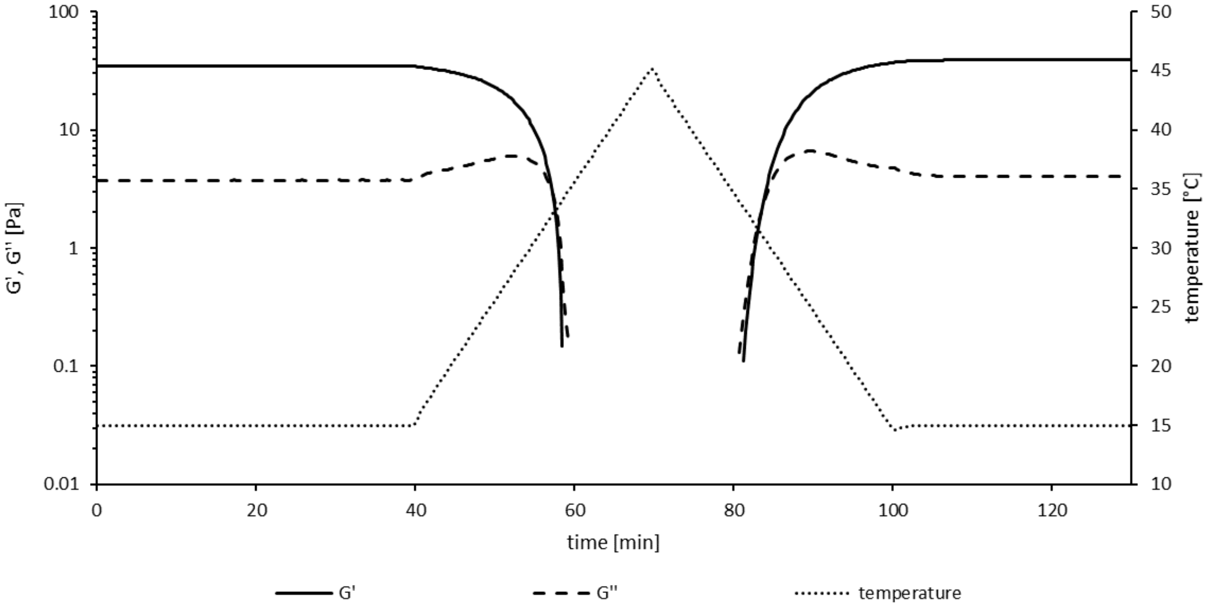

Figure S3: Variation of *G'* and *G"* with respect to the temperature for dirhamnolipid ethyl ester **2** in toluene (1 wt %) showing the reversibility of the gelation process [*A* = 0.5%, *f* = 1 rad/s].

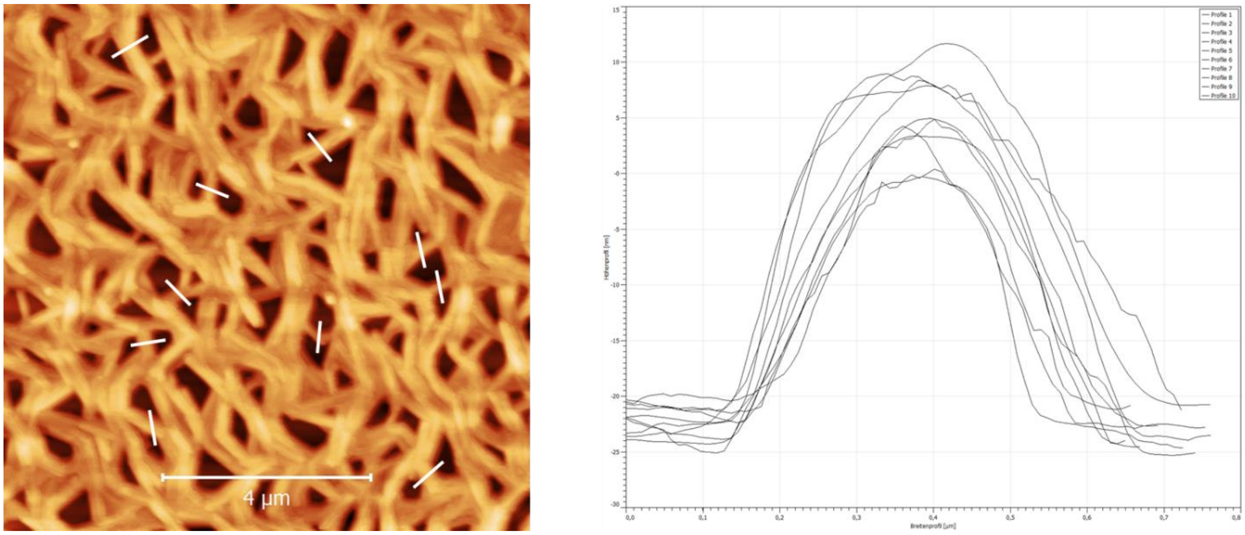

Figure S4: AFM height image and height profiles of the dirhamnolipid ethyl ester **2**/toluene (50 µM) system.

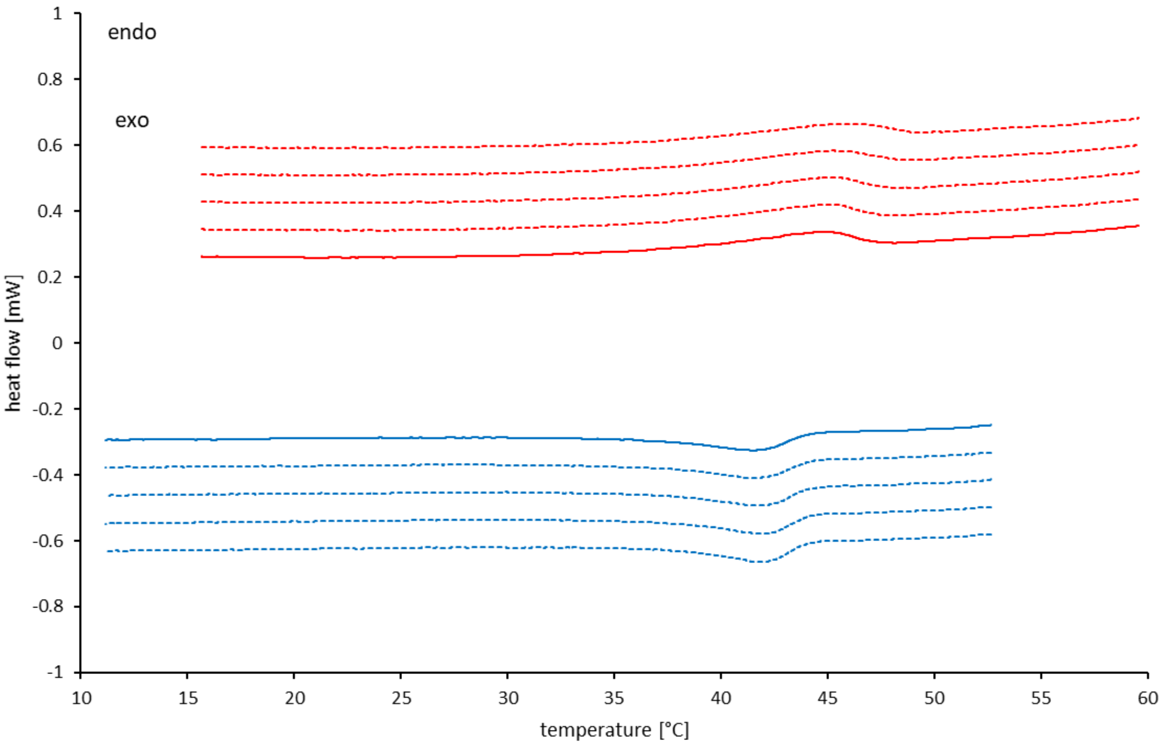

Figure S5: DSC (endo up) for **7** in toluene (5 wt %) with a heating/cooling rate of 10 K/min (continuous line, red = heating, blue = cooling). Further cycles are illustrated as dotted lines and are shifted by 0.1 mW with respect to the ordinate for the sake of visuality.

Table S2: DSC data obtained from **7** in toluene (5 wt %).

	heating		cooling	
**entry**	T_{GS} [°C]	ΔH_{GS} [J/g]	T_{SG} [°C]	ΔH_{SG} [J/g]
**1**	44.82	0.25	41.56	-0.24
**2**	44.92	0.25	41.74	-0.25
**3**	45.02	0.25	42.03	-0.25
**4**	45.14	0.26	42.23	-0.27
**5**	45.58	0.26	42.02	-0.26
∅	45.10	0.25	41.92	-0.25

3. Viscosity

The absolute value of the complex viscosity $|\eta^*|$ is related to the dynamic moduli and the shear frequency by the relation [1]:

$$|\eta^*| = \sqrt{\left(\frac{G'}{\omega}\right)^2 + \left(\frac{G''}{\omega}\right)^2}$$

The relationship between the complex viscosity and the shear frequency for the different concentrations of **2** in toluene at 15 °C is shown in Figure S6. The “shear-thinning” effect of non-Newtonian fluids is observed for all samples. This is a characteristic behavior of rodlike and wormlike micelles [2]. The linear decline of the viscosity can be explained by the reorientation of the micelles. With increasing shear frequency the micelles align themselves in the direction of increasing shear strain and produce less resistance. The viscosity levels out at high frequencies until all micelles are fully aligned. The fitted curve for the linear decline of the viscosity in the medium frequency range (Figure S6) corresponds well to the two parametric power law of Ostwald and de Waele [3]:

$$|\eta^*| = k \cdot \omega^{n-1}$$

where *k* is the flow consistenxy index ($Pa \cdot s^n$) and *n* is the dimensionless flow behavior index. Very small values were found for *n* (Table S3). Therefore, the complex viscosity is almost antiproportional to the shear frequency $|\eta^*| \sim \omega^{-1}$. The viscosity approaches a finite value at low frequency, which is called the zero-shear viscosity η_0. For Maxwell behavior η_0 can be obtained from the plateau modulus and the relaxation time ($\eta_0 = G_0 \cdot \tau_R$). The calculated values for η_0 (Table S3) match the experimental data for the complex viscosity at low frequency (Figure S6).

Table S3: Values of k, n, R^2, and η_0 of the dirhamnolipid ethyl ester **2**/toluene system.

conc. [% w/w]	*k*	*n*	R^2	η_0 [Pa·s]
0.5	1.135	0.072	0.9985	24.4
2.0	13.785	0.060	0.9998	393.0
3.0	25.127	0.058	0.9997	692.0

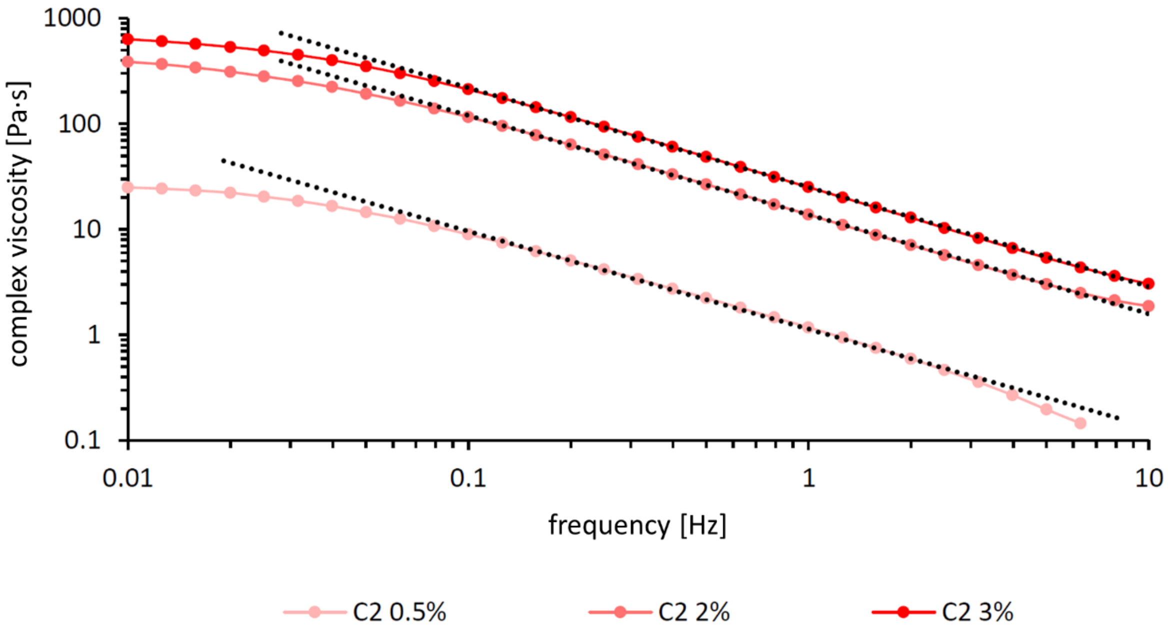

Figure S6: Plot of the complex viscosity |η*| against the shear frequency for different concentrations of **2** in toluene at 15 °C. The linear fit for the frequency values of 0.1–3 Hz is stated as dotted lines.

4. ^{1}H NMR and ^{13}C NMR spectra of the new compounds

Dirhamnolipid ethyl ester **2** ^{1}H: 400 MHz, ^{13}C: 101 MHz, solvent: $CDCl_3$.

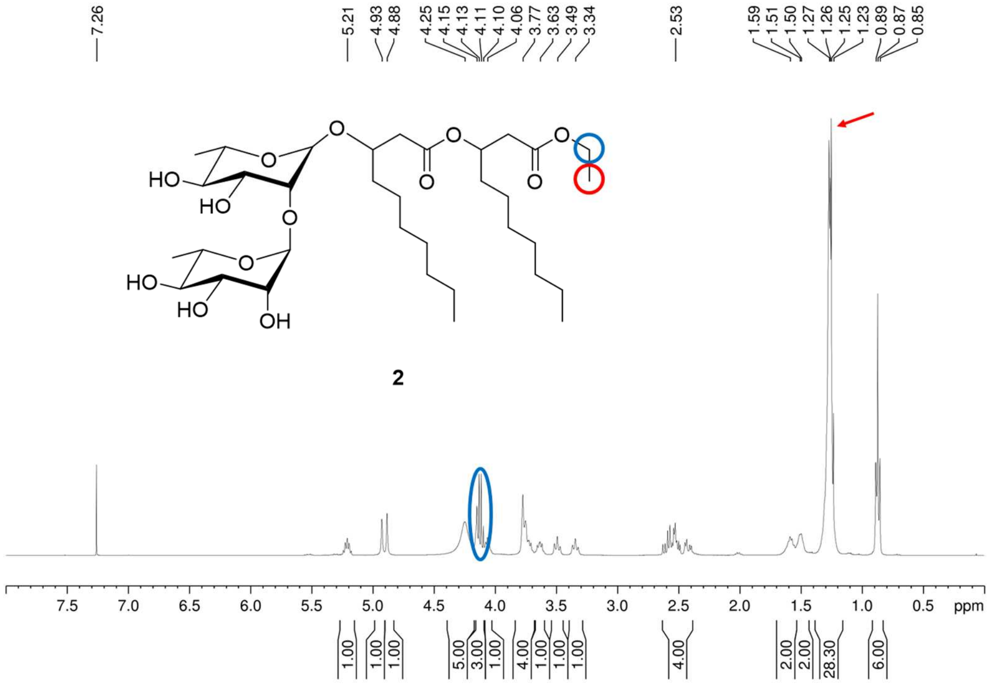

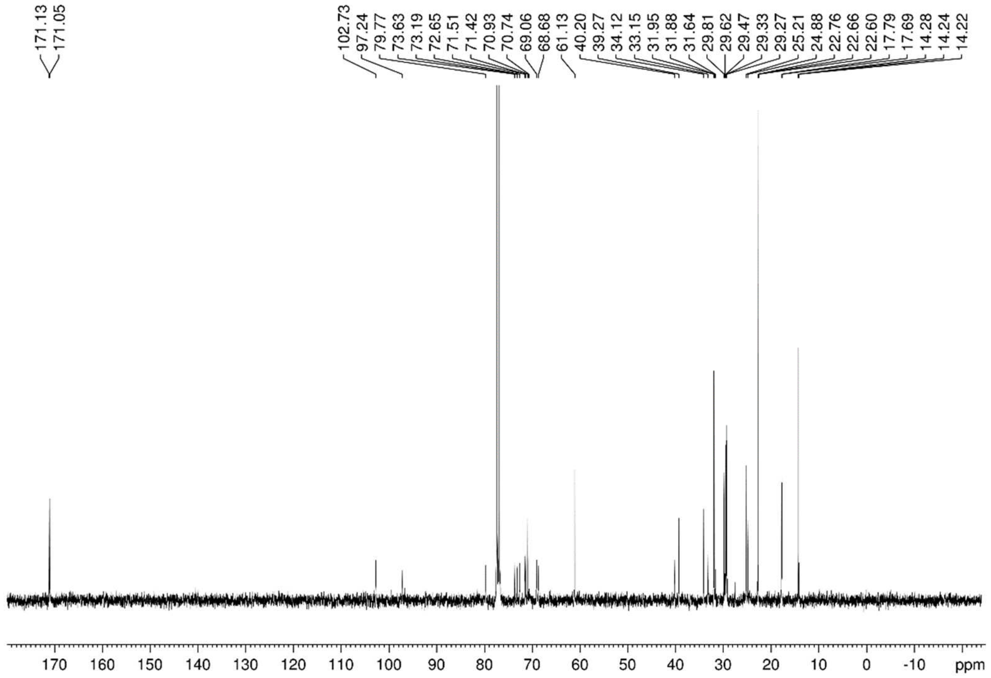

Dirhamnolipid octyl ester **3** ^{1}H: 600 MHz, ^{13}C: 151 MHz, solvent: $CDCl_3$.

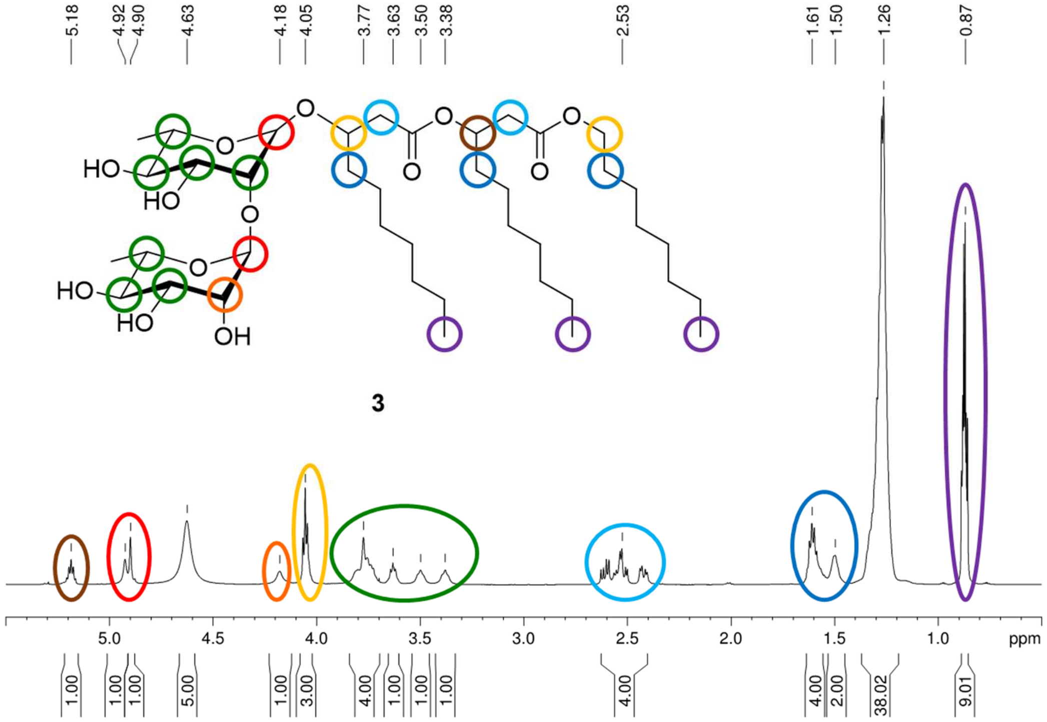

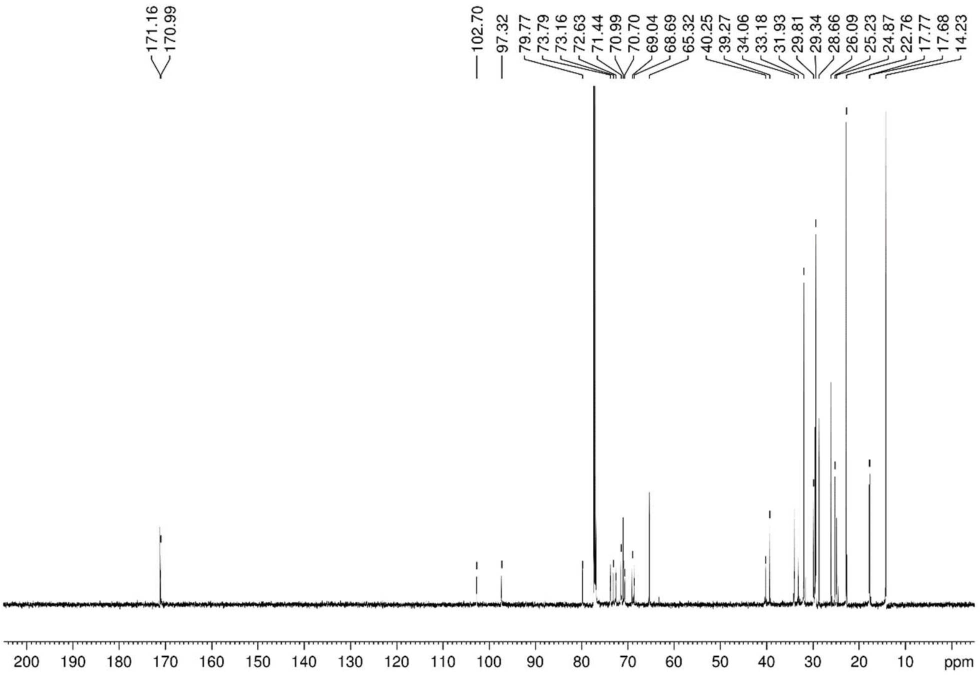

Dirhamnolipid hexadecyl ester **4** ^{1}H: 300 MHz, ^{13}C: 151 MHz, solvent: $CDCl_3$.

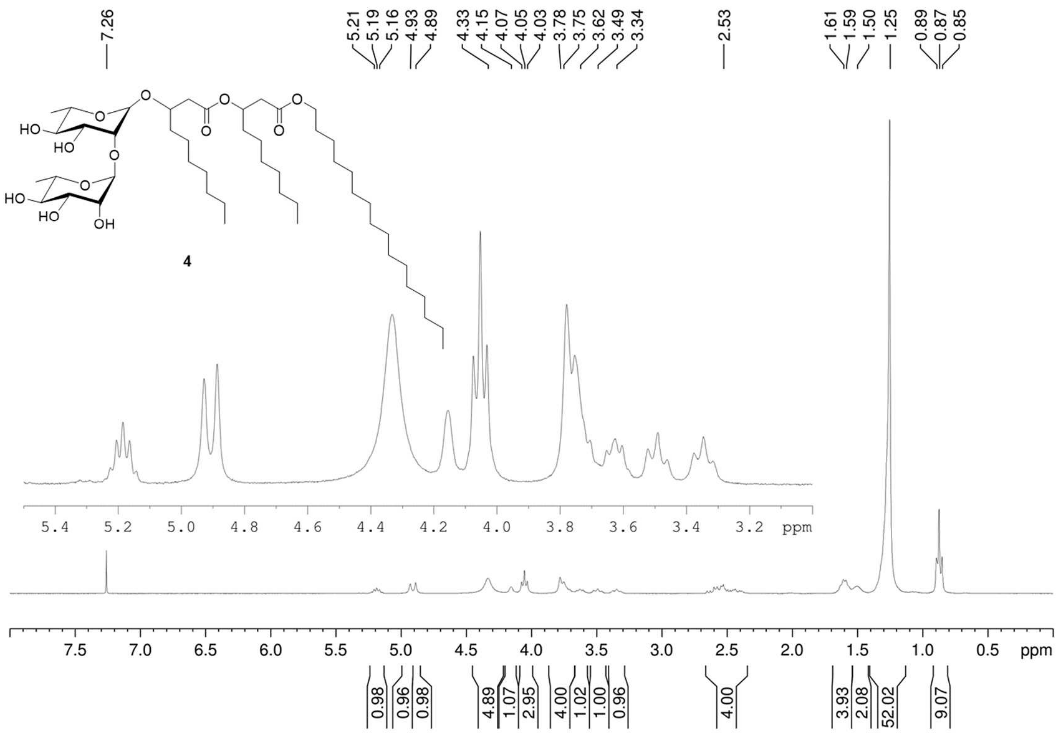

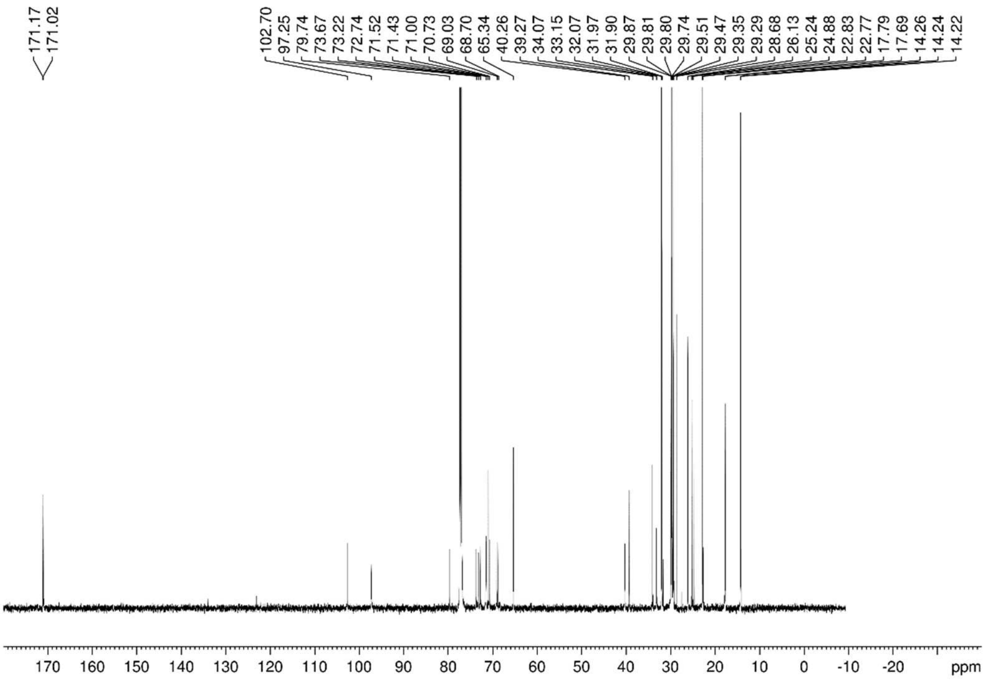

Dirhamnolipid octadecyl ester **5** ^{1}H: 300 MHz, ^{13}C: 151 MHz, solvent: $CDCl_3$.

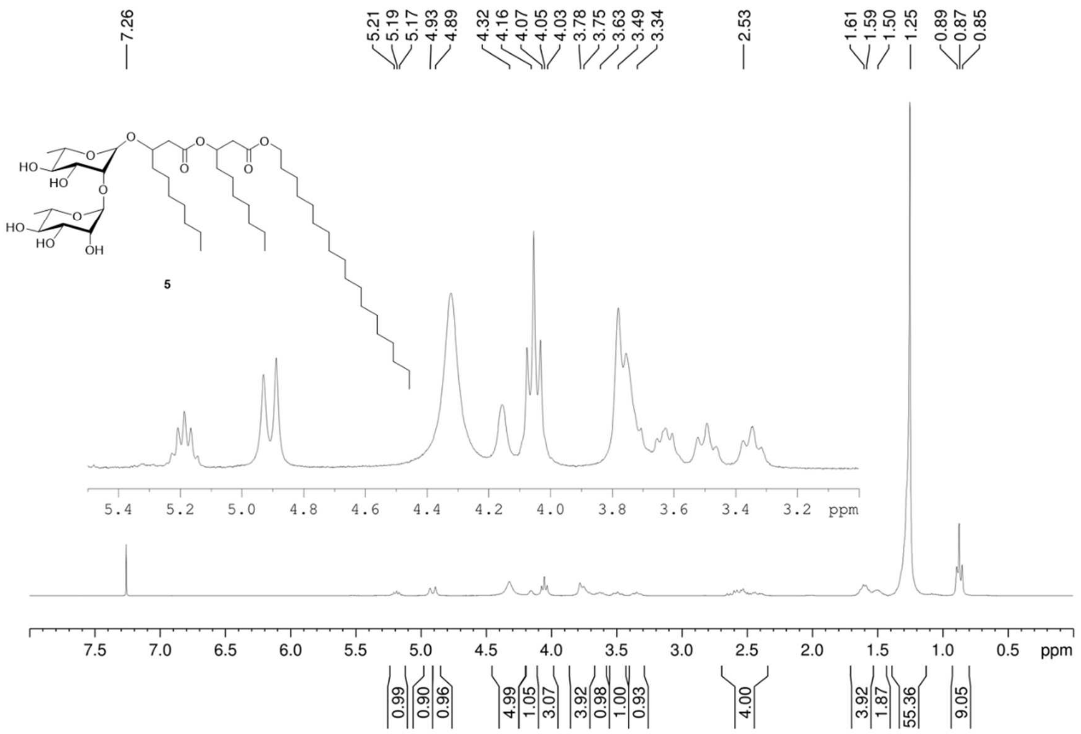

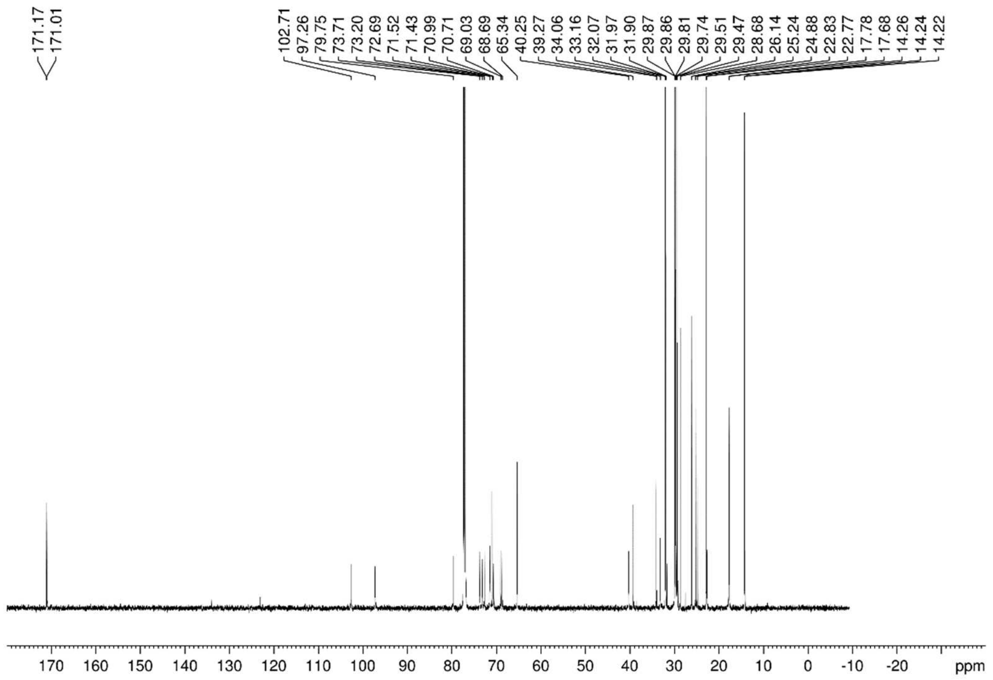

Dirhamnolipid docosyl ester **6** ^{1}H: 400 MHz, ^{13}C: 151 MHz, solvent: $CDCl_3$.

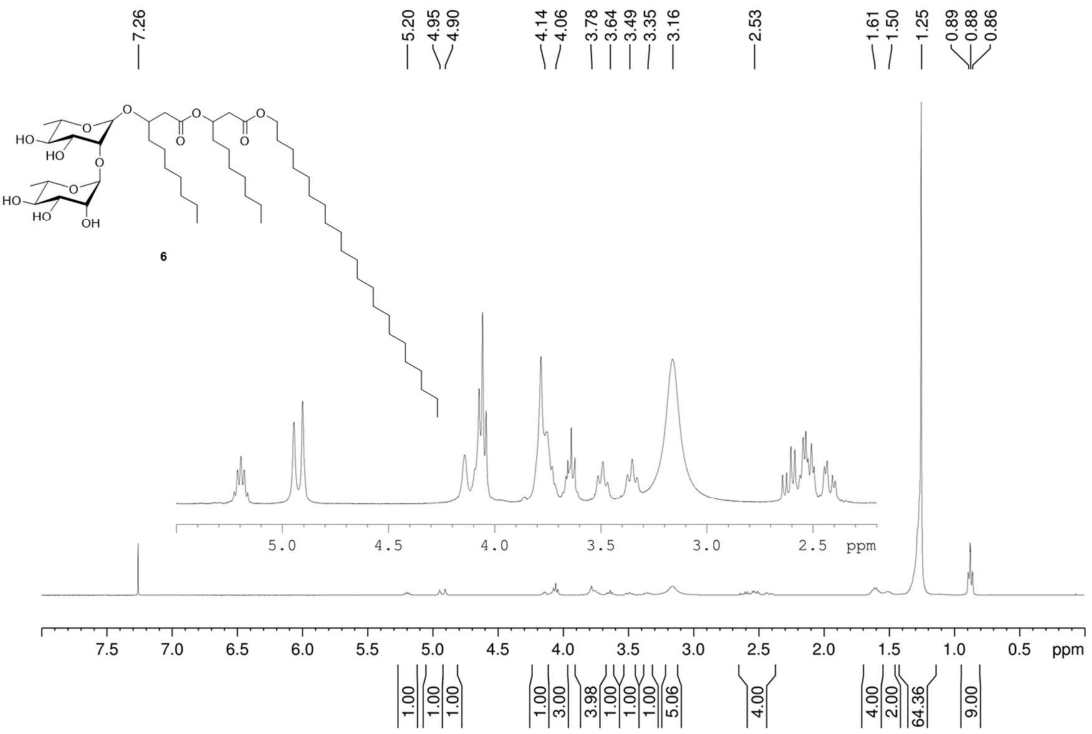

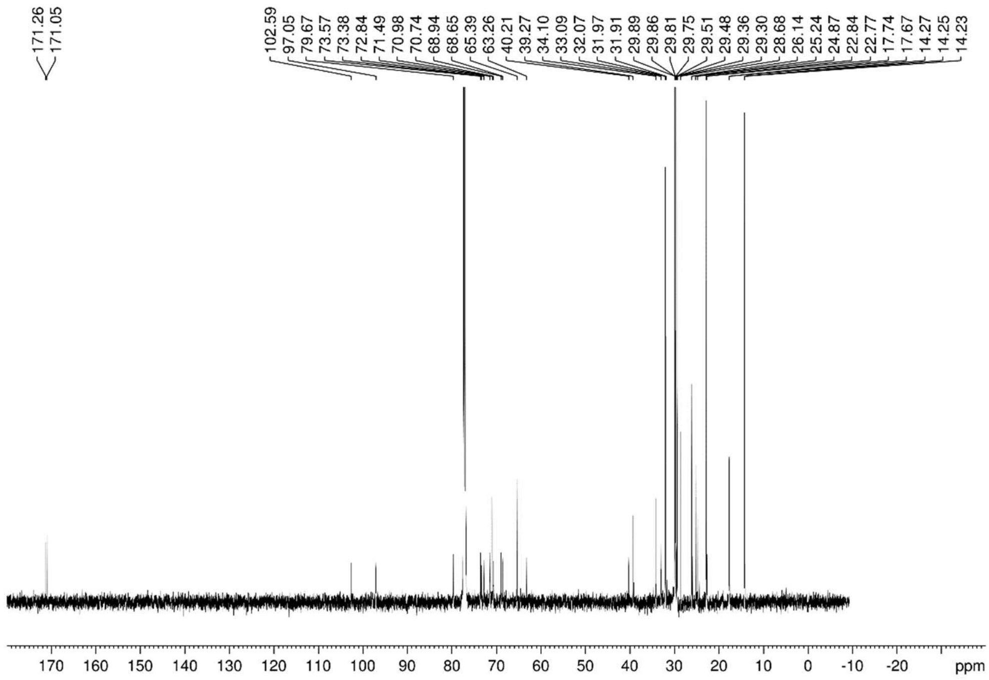

Dirhamnolipid-C6-dirhamnolipd **7** ^{1}H: 300 MHz, ^{13}C: 151 MHz, solvent: $CDCl_3$.

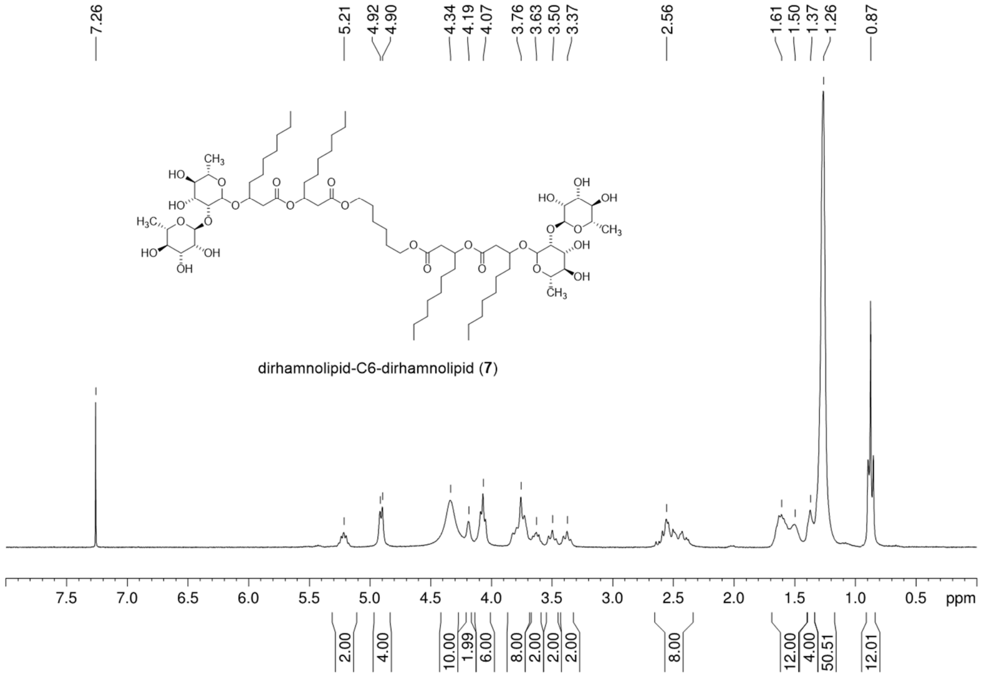

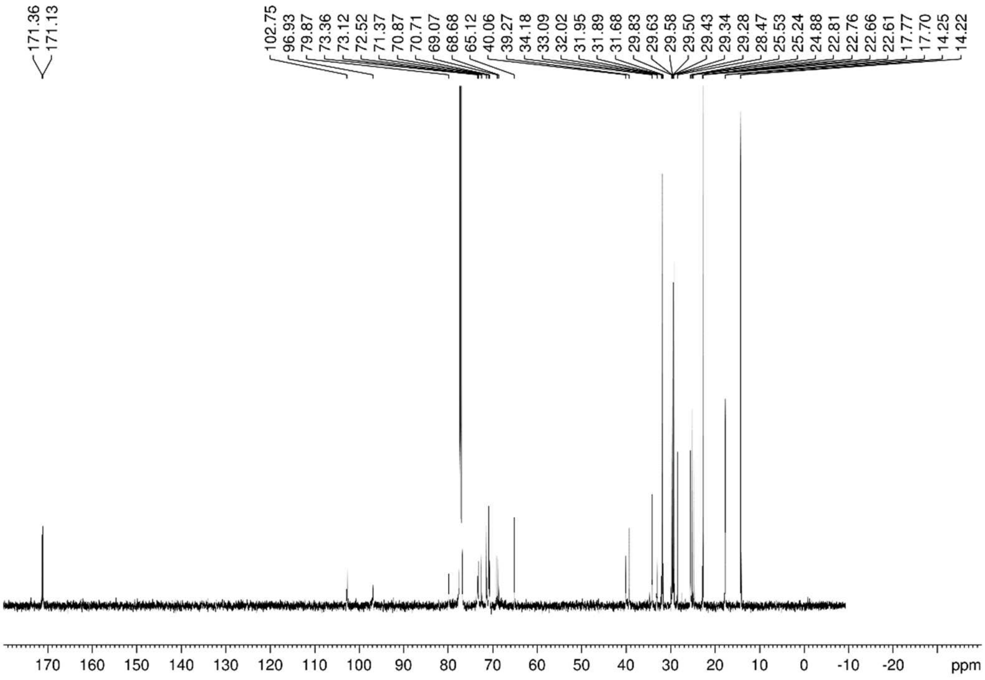

5. References

1. Shchipunov, Y. A.; Hoffmann, H. *Rheol. Acta,* **2000,** *39,* 542–553.

2. Rehage, H.; Hoffmann, H. *J. Phys. Chem.,* **1988,** *92,* 4712–4719.

3. Ostwald, W. *Colloid. Polym. Sci.,* **1929,** *47,* 176–187.
